# Ion specific effects on the immobilisation of charged gold nanoparticles on metal surfaces†

**DOI:** 10.1039/c7ra10374c

**Published:** 2018-01-05

**Authors:** C. Kaulen, U. Simon

**Affiliations:** JARA – FIT, RWTH Aachen University 52074 Aachen Germany corinna.kaulen@ac.rwth-aachen.de; Institute of Inorganic Chemistry, RWTH Aachen University 52074 Aachen Germany

## Abstract

Since the pioneering work of F. Hofmeister, *Arch. Exp. Pathol. Pharmakol.*, 1888, **24**, 247, ion specific effects have been steadily reported in the context of colloidal or protein stabilisation in electrolyte solutions. Although the observed effects are omnipresent in chemistry and biology, their origin is still under ferocious discussion. Here, we report on ion specific effects affecting the self-assembly of amine and carboxylic acid functionalised gold nanoparticles on metal surfaces as well as in electrolyte solution as a function of the monovalent cations Li^+^, Na^+^, K^+^ and Cs^+^. Mercaptooctanoic acid and 1,8-amine-octanethiol functionalised gold nanoparticles were adsorbed on structured AuPd/Pt substrates under addition of the respective chloride salts. Furthermore, the influence of the same salts on the salt induced aggregation of these AuNP was investigated. Our results demonstrate that the assembly processes on the metal surface as well as in electrolyte solution are influenced by the addition of different cations. We attribute the observed effects to ion pairing of the functional end groups with the added cations. With these findings we introduce a new parameter to control the self-assembly of 2D AuNP arrays on solid supports or of 3D AuNP networks in solution, which could be of relevance for the fabrication of new tailor-made functional materials or for biomedical applications.

## Abstract 2

Different monovalent cations influence the immobilisation of carboxylic acid and amine terminated gold nanoparticles.

## Graphical abstract

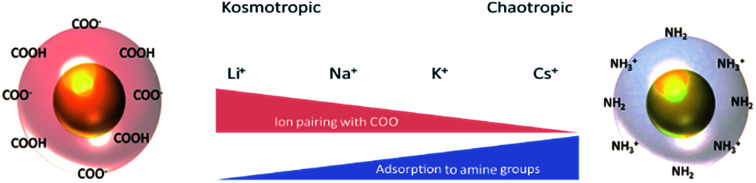

## Introduction

**Fig. 1:**
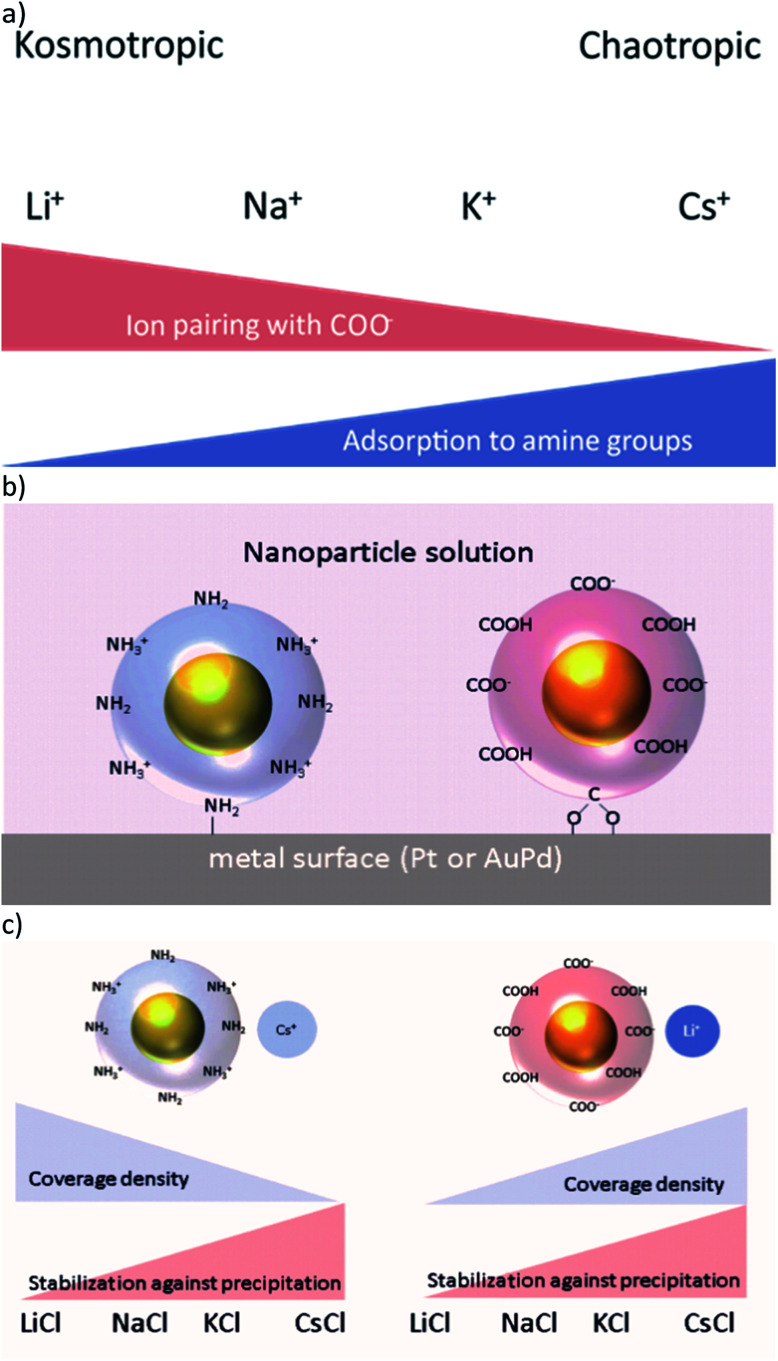
(a) Kosmotropic cations form strong ion-pairs with COO^−^ groups, chaotropic ions adsorb to neutral NH_2_-groups. (b) Proposed binding model: adsorption to the metal surface by coordinative bonds of the AuNPs' end groups. (c) For Au-AOT decreasing coverage density and increasing stability against precipitation is expected for more chaotropic cations, while for Au-MOA an inverse trend in coverage density and similar stability against precipitation is expected.

Hofmeister was the first to describe protein precipitation influenced by different added salts in 1888.^1^ From his experiments he ranked a bundle of commonly used cations and anions according to their effect on the precipitation of proteins. Later on, ions were named according to their ability to structure water, *i.e.* ions with a strongly bound water shell are classified as kosmotropic and ions with a weak hydration shell are called chaotropic.^2^ Among the monovalent cations Li^+^ and Na^+^ are kosmotropic, whereas K^+^, Rb^+^, and Cs^+^ are chaotropic (Fig. 1a). Until now, numerous experimental observations have been reported, in which the nature of the applied salts has a crucial influence.^3–5^ These findings comprise the stabilisation of colloids,^3,6–8^ the formation of micelles,^4,9^ and the interfacial structure of charged surfaces.^5,10,11^ The origin of these ion specific effects is still unclear, although several attempts at an explanation have been made.^12,13^

Our long term goal is to integrate ligand stabilised gold nanoparticles (AuNPs) by self-assembly on electrode surfaces, in a directed manner, thus building up hybrid nanoelectronic devices with advanced functionality.^14,15^ In this context, we presented in our previous work the directionally controlled immobilisation of tailored ligand stabilised Janus-AuNPs on gold surfaces, prefunctionalised with self-assembled monolayers (SAMs) of thiol molecules.^16^ Thereby, we took advantage of electrostatic and hydrophilic/hydrophobic interactions. Beyond this, we investigated the differential adsorption of ligand stabilised AuNP on pristine gold (alloyed with 30% palladium due to the manufacturing process) and platinum surfaces depending on the pH and the ionic strength of the immobilisation solution.^17^ We found that mercaptooctanonic acid (MOA) functionalised AuNPs (Au-MOA) at pH 9 and 1,8-aminooctanethiol (AOT) stabilised AuNPs (Au-AOT) at pH 3 adsorb on platinum surfaces with high selectivity (Fig. 1b). Further, we could optimise the selectivity and the coverage density of adsorbed AuNPs by adjusting the ionic strength of the immobilisation solution. Following the theory of ion specific effects, interactions of different monovalent cations with charged (–COO^−^/–NH_3_^+^) and uncharged (–COOH/–NH_2_) end groups of the AuNPs should influence the covering density of the particles on metal surfaces as well as their salt induced precipitation (Fig. 1c).^7,8^ Based on these considerations, herein we present a detailed study on the adsorption characteristics of amine terminated Au-AOT and carboxylic acid terminated Au-MOA on pristine AuPd and Pt surfaces under the influence of different monovalent chloride salts MCl (M = Li^+^, Na^+^, K^+^, Cs^+^). We chose AuPd and Pt as metal surfaces to determine the binding affinity of amine and carboxylic acid groups to these technically relevant electrode materials. Furthermore, we evaluate the colloidal stabilities of the Au-MOA and Au-AOT solutions with addition of the respective salts MCl by UV-Vis spectroscopy and we analyse the formed aggregates by SEM. Finally, the results of these investigations could be applied usefully on the self-assembly of AuNP for the construction of metamaterials,^18^ sensing of biomolecules^19,20^ or the formation of tailor-made AuNP aggregates for theranostics.^21^

## Experimental

### Materials

The following chemicals were purchased from Sigma-Aldrich Chemie GmbH and were used as received: hydrogen tetrachloroaurate(iii) trihydrate (HAuCl_4_·3H_2_O), trisodium citrate dihydrate (C_6_H_5_Na_3_O_7_·2H_2_O), MOA, 4-(2-hydroxyethyl)-1-piperazine-ethanesulfonic acid (HEPES), tris(hydroxymethyl)aminomethane (TRIS), and 3-triethoxysilylpropylamine (APTES). Glass beads were purchased from Carl Roth GmbH + Co. KG. AOT was purchased from Dojindo Molecular Technologies, Inc. All glassware was cleaned with aqua regia and rinsed with copious amount of water prior to use. Ultrapure water with a conductivity <55 nS cm^−1^ was used for all procedures.

### Instrumentation and particle characterisation

UV-Vis absorption spectra were recorded with a JASCO V-630 spectrophotometer, dynamic light scattering (DLS) measurements were performed with a Malvern Zetasizer Nano S, He–Ne-laser *λ* = 633 nm, *P* = 4 mW, *θ* = 173° in order to determine the hydrodynamic radii of single AuNP and AuNP agglomerates, respectively, Scanning Electron Microscopy (SEM) was conducted with a high resolution Field Emission Scanning Electron Microscope (FE-SEM, LEO/ZEISS Supra 35 VP, Oberkochen, Germany) and, particle concentrations were estimated from gold concentrations determined by atomic absorption spectroscopy (AAS, Shimadzu AA-7000, Tokyo, Japan).

### Substrate fabrication

Structured Pt/AuPd substrates were fabricated as previously described.^17^ Briefly, a Si (100) substrate (1 × 1 inch, 1–5 Ω cm^−1^) was covered with an insulating SiO_2_ layer (430 nm) which was created by oxidation of Si using thermal dissociation of water vapour at 1500 °C for 50 min. Subsequently, a full plain metallic layer was fabricated by vapour deposition of titanium (adhesion layer, 10–20 nm) and platinum (100 nm, deposition rate 2 nm s^−1^, pressure <1 × 10^−7^ mbar) onto the Si/SiO_2_ substrate. Thin AuPd (30% Pd) lines with a thickness of 15 nm and with varying widths between 500 nm and 1 μm were defined *via* e-beam lithography in a lift-off process on top of the platinum layer. After lift-off the sample was rinsed with 2-propanol and then dried in a stream of N_2_.

### Adsorption studies

The Pt/AuPd substrates were cut into 5 × 5 mm^2^ pieces. The substrate pieces were washed with 5 mL ethanol and subsequently treated with oxygen plasma (0.4% O_2_, 100 W) for 2 minutes in order to remove organic residues. 10 μL of the Au-MOA solution in HEPES/TRIS buffer (10 mM) at pH 9 (particle concentration of 9.6 × 10^−9^ mol L^−1^) or the Au-AOT in diluted HCl at pH 3 (particle concentration of 4.4 × 10^−9^ mol L^−1^) was drop cast onto the cleaned surface, immediately after plasma treatment. The sample was kept in a H_2_O saturated atmosphere to prevent evaporation of solvent. After 20 min., the substrate was rinsed with water in order to remove unbound AuNPs and dried in a nitrogen stream. The substrates were analysed by SEM and statistical evaluations of the particle density were performed related to an area of 1 μm^2^ on 9 different positions of the substrate.

### Colloidal stability studies

Au-MOA and Au-AOT were synthesized and purified as previously described.^14^ After the last centrifugation step Au-MOA were redispersed in 10 mM HEPES/TRIS buffer at pH 9 and the required amount of a 1 M MCl solution was added to final salt concentrations between 40 mM and 120 mM. The Au-MOA concentration was 4.5 × 10^−9^ mol L^−1^. Au-AOT were redispersed in 10^−3^ M HCl (pH 3) and the required amounts of 1 M MCl solutions were added to final salt concentrations of 100 mM to 250 mM. The Au-AOT concentration was 2.2 × 10^−9^ mol L^−1^. Immediately after addition of the salt solution UV-Vis spectra of the colloid solutions were collected within 3 minute intervals for one hour.

### SEM investigation of the formed aggregates

A Au-MOA solution (4.5 × 10^−9^ mol L^−1^) in HEPES/TRIS at pH 9 with 45 mmol NaCl and a Au-AOT solution (2.2 × 10^−9^ mol L^−1^) in 10^−3^ M HCl (pH 3) with 125 mmol NaCl were prepared. Immediately after mixing 10 μL samples were taken every five minutes and deposited on a carbon-coated TEM grid. After another five minutes, the solution was removed with a KIM wipe and the grids were allowed to dry at 20% air humidity and 20 °C. SEM images were acquired in transmission mode.

## Results and discussion

### Adsorption of Au-MOA and Au-AOT on Pt and AuPd surfaces in dependence of the addition of different monovalent chloride salts

**Fig. 2:**
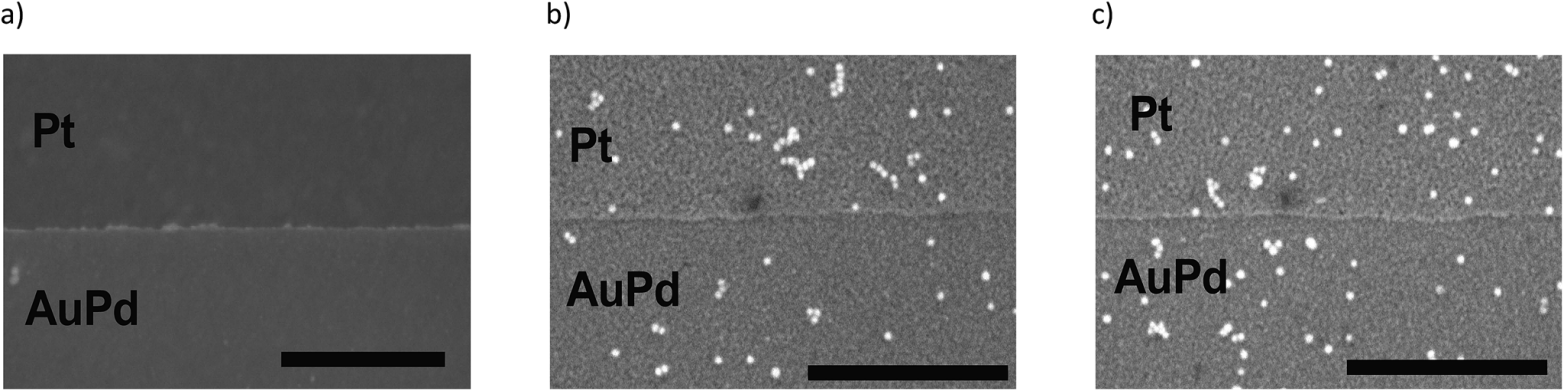
SEM images of adsorption experiments on substrates consisting of a 100 nm Pt layer with 15 nm AuPd structures on it without salt addition, (a) Au-MOA in HEPES/TRIS at pH 9; (b) Au-AOT in diluted acetic acid at pH 3; (c) Au-AOT in diluted HCl at pH 3. Scale bar represents 500 nm.

**Fig. 3:**
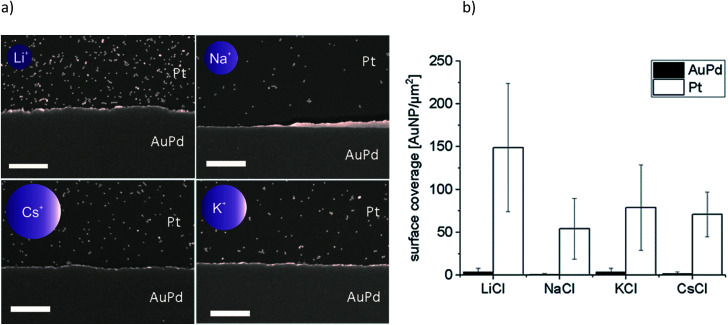
(a) Representative SEM images of adsorption experiments of Au-MOA with added monovalent salts MCl (M = Li, Na, K, Cs, the size of the schematic ions correlates to the sizes of the corresponding ionic radii) and (b) the determined covering densities, where the error bars show the mean of three experiments and the nine different spots which were analysed on each substrate, scale bar represents 500 nm.

Au-MOA nanoparticles with a mean diameter of 13.1 nm ± 1.3 nm were synthesized from citrate stabilised AuNPs by a ligand exchange reaction with MOA as previously published,^17^ purified by centrifugation and redispersed in HEPES/TRIS buffer at pH 9. For the synthesis of Au-AOT (mean diameter 14.9 nm ± 1.2 nm) a solid phase supported approach was applied in order to prevent irreversible aggregation of the citrate stabilised AuNPs during ligand exchange with AOT.^17^ After synthesis the Au-AOT were purified by centrifugation and redispersed in diluted HCl solution at pH 3. The corresponding UV-Vis spectra, the hydrodynamic diameter and zeta-potentials determined by DLS, and representative SEM images are displayed in the ESI (Fig. S1–S4 in the ESI†). A zeta-potential change from −69.8 ± 7.9 mV for citrate stabilised AuNP to −42.5 ± 9.8 mV at pH 9 for Au-MOA and to +60.7 ± 8.5 mV for Au-AOT at pH 3 clearly indicates successful ligand exchange. In a first set of experiments, we investigated the effect of the monovalent cations M = Li^+^, Na^+^, K^+^, and Cs^+^ on the adsorption characteristics of carboxylic acid terminated AuNPs (Au-MOA) on AuPd and Pt surfaces. We chose this row of monovalent cations, because the investigated Au-AOT and Au-MOA are stable at moderately enhanced electrolyte concentrations of these salts, while already very low concentrations of divalent salts like Mg^2+^ or Ca^2+^ lead to aggregation of Au-MOA.^22^ Therefore, neither, the influence of divalent cations, nor the impact of different monovalent anions was investigated, as Au-AOT showed oxidative degradation of the Au core upon addition of I^−^ or Br^−^.^23^ In order to transfer our results to experiments with nanoelectrode structures (nanogaps), we chose specially designed substrates, consisting of a layer of Pt (100 nm) with 1 μm broad AuPd (30% Pd, 15 nm) lines on it to enable a directed deposition of the functionalised AuNP *via* self-assembly.^17^ This design of the structured substrates ensures that the adsorption of the AuNPs on the AuPd and Pt surfaces takes place under identical conditions regarding temperature, pH and AuNP concentration. Samples were prepared by adding an aqueous solution of the respective MCl solution (M = Li^+^, Na^+^, K^+^, and Cs^+^) to each Au-MOA-HEPES/TRIS dispersion, whereby the added MCl amount was adjusted according to a final salt concentration of 10 mM. Additionally, a reference sample in pure HEPES/TRIS buffer was set up. 10 μL of the freshly prepared Au-MOA solutions were drop cast on the structured AuPd/Pt substrates. This kind of sample preparation was used to emulate the trapping of the AuNPs between nanostructured electrodes. During incubation the substrates were kept in a water saturated atmosphere and were gently swayed in order to avoid the so called “coffee ring effect”.^24^ After 45 min, the substrates were rinsed with water and dried in a nitrogen stream. SEM images were taken from nine equally distributed different spots on each substrate. Firstly, on the surface of the reference sample without any salt addition we could detect only single adsorbed particles (Fig. 2a). Which illustrates that salt addition is necessary to assist the adsorption process. Secondly, all images of the samples with addition of the different salts show moderate particle coverage on platinum but no adsorbed Au-MOA on the AuPd surface. The Au-MOA are present as single particles or small assemblies of 2–10 particles. The density of adsorbed AuNP (*Γ* Pt and *Γ* AuPd) was determined by counting the adsorbed AuNP within in each spot comprising an area of 1 μm^2^. Exemplary SEM images as well as respective particle surface covering densities are shown in Fig. 3 (see Fig. S5 in the ESI† for additional images).

These results reveal that the highest surface coverage density was determined in the presence of Li^+^ and the lowest in the presence of Na^+^. In the presence of K^+^ and Cs^+^ the amount of AuNP was in between the aforementioned. A certain variation in covering density was observed, which we assign to the concentration fluctuation of the AuNP solution during sample preparation. Nevertheless, the fluctuation does not affect the determined trend. Moreover, in all experiments we found preferential adsorption on the Pt surface, which portends that the mechanism of adsorption changes not substantially with addition of the different cations.

**Fig. 4:**
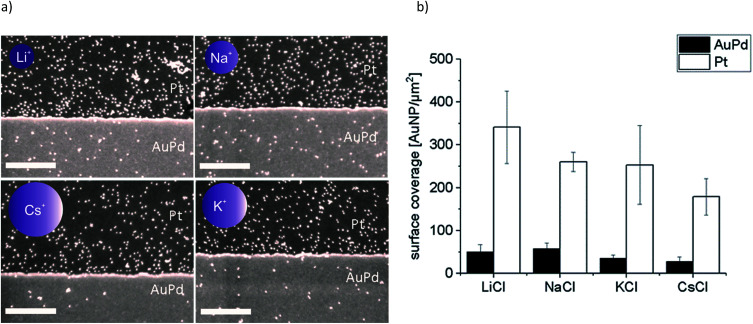
(a) Representative SEM images of adsorption experiments of Au-AOT with added monovalent salts MCl (M = Li, Na, K, Cs, the size of the schematic ions correlates to the sizes of the corresponding ionic radii) and (b) the determined covering densities, where the error bars show the mean of three experiments and the nine different spots which were analysed on each substrate, scale bar represents 500 nm.

In a second set of experiments we investigated the adsorption of Au-AOT in the presence of the same row of chloride salts MCl (M = Li, Na, K, Cs). An aqueous solution of the respective MCl solution was added to each Au-AOT dispersion, whereby the added MCl amount was adjusted accordingly to a final salt concentration of 10 mM. Here, we also provided a reference sample without MCl addition. Beyond this, we prepared a chloride free Au-AOT solution by redispersing the Au-AOT in diluted acetic acid, to understand the influence of the chloride on the adsorption process. 10 μL of the freshly prepared Au-AOT solutions were drop cast on the structured AuPd/Pt substrates and the substrates were prepared for SEM analysis in the same way as for the Au-MOA. The SEM images of the reference samples are displayed in Fig. 2b and c. On both reference samples Au-AOT are adsorbed with low covering densities on either Pt or AuPd surfaces with no specific preference. Fig. 4 shows the respective SEM images of the adsorption experiments of Au-AOT with added MCl as well as the evaluated coverage densities (see Fig. S6 in the ESI† for further images). Here a sub-monolayer of particles had formed on the Pt surface while several single Au-AOT were present on the AuPd surface. We evaluated the covering densities for amine terminated AuNP on Pt and AuPd as described for Au-MOA. This evaluation revealed decreasing AuNP density on Pt in the following order: Li > Na ≈ K > Cs. Overall, the Au-AOT surface coverage densities on AuPd were significantly lower than on Pt, thus pointing out that there is no influence of the different cations on the selectivity of the adsorption. However, our results reveal that there is indeed an influence of the different cations on the amount of adsorbed AuNPs. In order to explain the observed effect, we took into account (i) the influences of salt ions on the AuNPs' end-groups (Fig. 1c) and (ii) the proposed binding model of the AuNP on the metal surface (Fig. 1b).

Concerning (i), we expected ion pairing of kosmotropic cations with the charged carboxylate groups of Au-MOA and charge compensation by adsorption of cations to neutral carboxylic acid groups. The same holds for the amine end groups of Au-AOT, hence there will be ion pairing of chloride anions with positively charged ammonium groups as well as adsorption of the different cations to the neutral amine groups in various extent at the AuNPs' end groups. The number of charged end groups on AuNP stabilized by a SAM with pH sensitive groups like carboxylic acid or amine groups, depends on the pH of the solution. As pointed out by Grzybowski *et al.*, the p*K*_a_ of carboxylic acid groups rises significantly when the groups are densely packed within a SAM on a gold surface.^25^ Thus, the p*K*_a_ of the carboxylic acid groups on Au-MOA lies between the p*K*_a_ of free MOA molecules in solution (p*K*_a_ ∼ 4.8) and the p*K*_a_ of MOA within a monolayer on a flat gold surface (p*K*_a_ ∼ 10).^26^ That implies that at pH 9 the ligand shell of the Au-MOA exists of neutral carboxylic acid end groups and of negatively charged carboxylate end groups. The same applies to the amine end groups of Au-AOT at pH 3 hence, there will be neutral amine groups as well as positively charged ammonium groups on the AuNP surface (Fig. 1b).

Concerning (ii), if adsorption of AuNPs occurs *via* coordinative binding of the terminal end groups and the metal surface, we expect highest covering densities, when many terminal groups with free electron pairs are available. *i.e.* when no ion pairing of the –COO^−^ and the –NH_2_ groups with ions takes place.

To understand the interactions of the charged end groups with ions we investigated the aggregation behaviour of the Au-AOT and Au-MOA in the respective electrolyte solutions. Salt induced aggregation occurs by depletion of the electrochemical double layer surrounding the particles in aqueous solution.^27^ Aggregation starts when the charges of the end groups are shielded due to the formation of ion-pairs with the available ions in solution and thereby the electrostatic repulsion minimizes, so that particle collisions lead to precipitation. This should lead to stabilisation of Au-MOA and Au-AOT in electrolyte solutions with chaotrope cations (Fig. 1c). Therefore, we performed salt induced aggregation experiments in order to evaluate the interactions of the respective terminal group (–COOH or –NH_2_) with the particular M^+^ cations (M^+^ = Li^+^, Na^+^, K^+^, Cs^+^).

### Time dependent aggregation of Au-MOA and Au-AOT upon addition of different salts MCl (M = Li, Na, K, Cs)

**Fig. 5:**
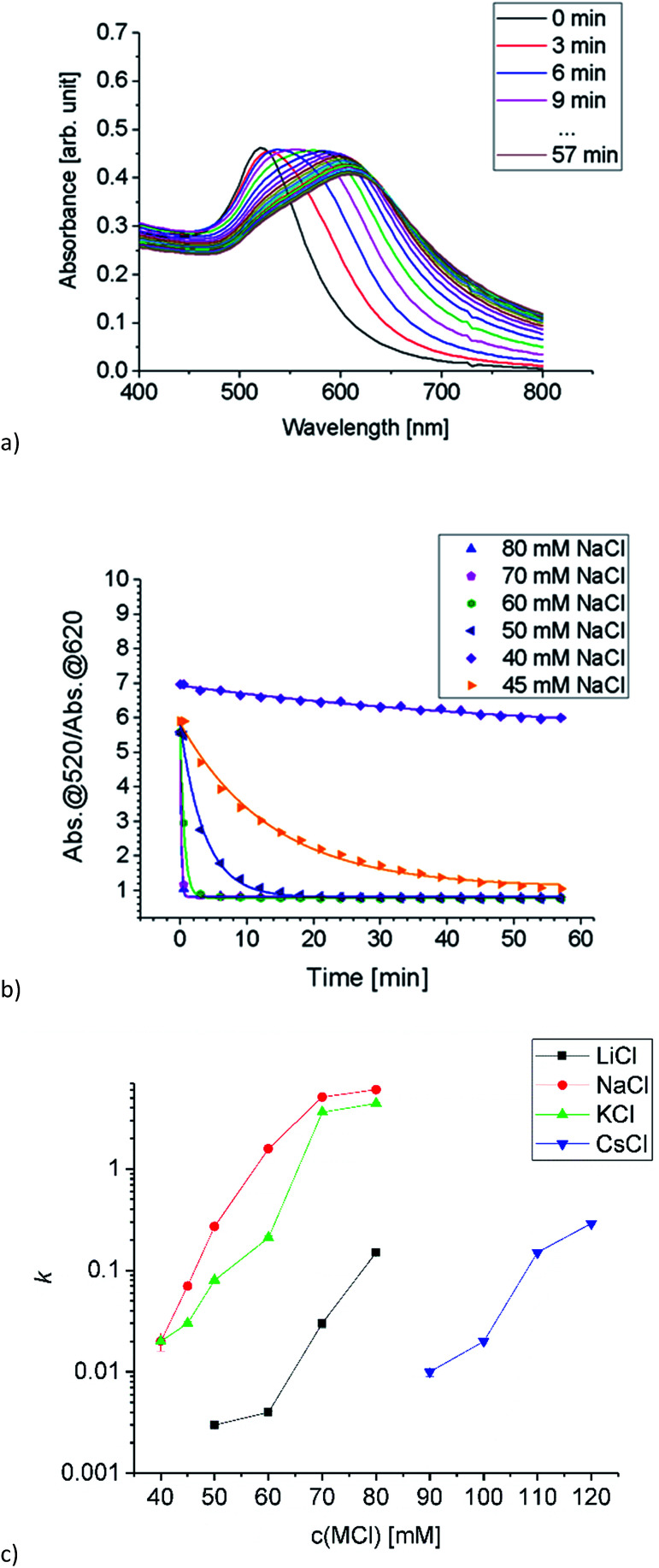
(a) Time dependent absorption spectra of Au-MOA in HEPES/TRIS at pH 9 after adding NaCl (final concentration 50 mM), spectra were taken every 3 minutes over 1 h. (b) Progression of the ratio of absorbance *R* (symbols) for NaCl concentrations from 40 mM to 80 mM and the respective fit curves (lines) fitted by a first order exponential decay function. (c) Semi-logarithmic plot of the aggregation constant *k* as a function of electrolyte concentration for the time dependent aggregation of Au-MOA. The higher the value of *k*, the faster the aggregation proceeds.

A stock solution of Au-MOA in HEPES/TRIS buffer at pH 9 was prepared. Samples were taken from these stock solution and different amounts of a 1 M MCl solution were added leading to final salt concentrations from 40 mM to 250 mM. Immediately after mixing of the sample, aggregation was observed by UV-Vis spectroscopy. Additionally, we took for one representative experiment, namely the aggregation with 45 mmol NaCl, every five minutes samples and deposited them on TEM grids in order to determine the shape and size of the formed aggregates. The SEM images illustrate quite vivid that within five minutes aggregates with a size of up to 100 nm are formed and after 15 minutes larger up to 500 nm long and 100 nm broad, most elongated aggregates dominate (see Table 1 in the ESI†). These aggregates lead to a significant red shift of the AuNPs' plasmon resonance. While single AuNPs show a maximum plasmon resonance at 520 nm, aggregates absorb at higher wavelengths. Therefore, to determine the ratio of single particles to aggregates the ratio *R* of absorbance at 520 nm to the absorbance at 620 nm was calculated and plotted as a function of time.^23,27–29^ The UV-Vis spectra of the aggregation experiment of Au-MOA with NaCl and the derived time-dependent course of the ratio *R* of absorbance are depicted in Fig. 5a and b. All UV-Vis spectra and the aggregation curves of Au-MOA with LiCl, NaCl, KCl, and CsCl are given in the ESI (Fig. S7–S31†). In order to compare the stability of the AuNPs in the different salt solutions, the aggregation curves were fitted by a first order exponential decay function (eqn (1)). From this fit curves the rate constants of the aggregation were calculated by *k* = *t*^−1^.1
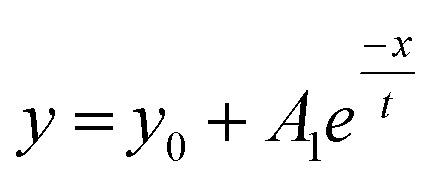


The derived *k* values are plotted as a function of electrolyte concentration (Fig. 5c), where values close to zero reflect slow aggregation, while values close to 1 and higher express fast aggregation. Based on these results the stability of Au-MOA is highest in CsCl, reduced in LiCl and lowest in KCl and NaCl. That means, the terminal carboxylate group forms strong ion pairs with Na^+^ and K^+^, while solvent separated ion pairs are formed with Li^+^ and Cs^+^. According to the theory of ion specific effects, the carboxylic acid group is considered as a hard anion, which is expected to form strong ion pairs with hard cations like Li^+^ and Na^+^. This correlates with our observations for Na^+^ but Li^+^ seems to act more like a soft cation. One explanation for this phenomenon can be derived from the fact that the aggregation of the Au-MOA also occurs by charge compensation due to adsorption of cations to the neutral carboxylic acid groups: the adsorption of Li^+^ at the neutral COOH groups is hindered by the strong hydration shell of Li^+^, thus preventing charge compensation of the overall negatively charged Au-MOA and thereby hampering salt induced aggregation, as shown also on COOH terminated SAMs by MD simulations.^30,31^ Further, a comparable exception is reported for ion pairing of monovalent salts with carboxylate groups of acetate and glycine.^32^

**Fig. 6:**
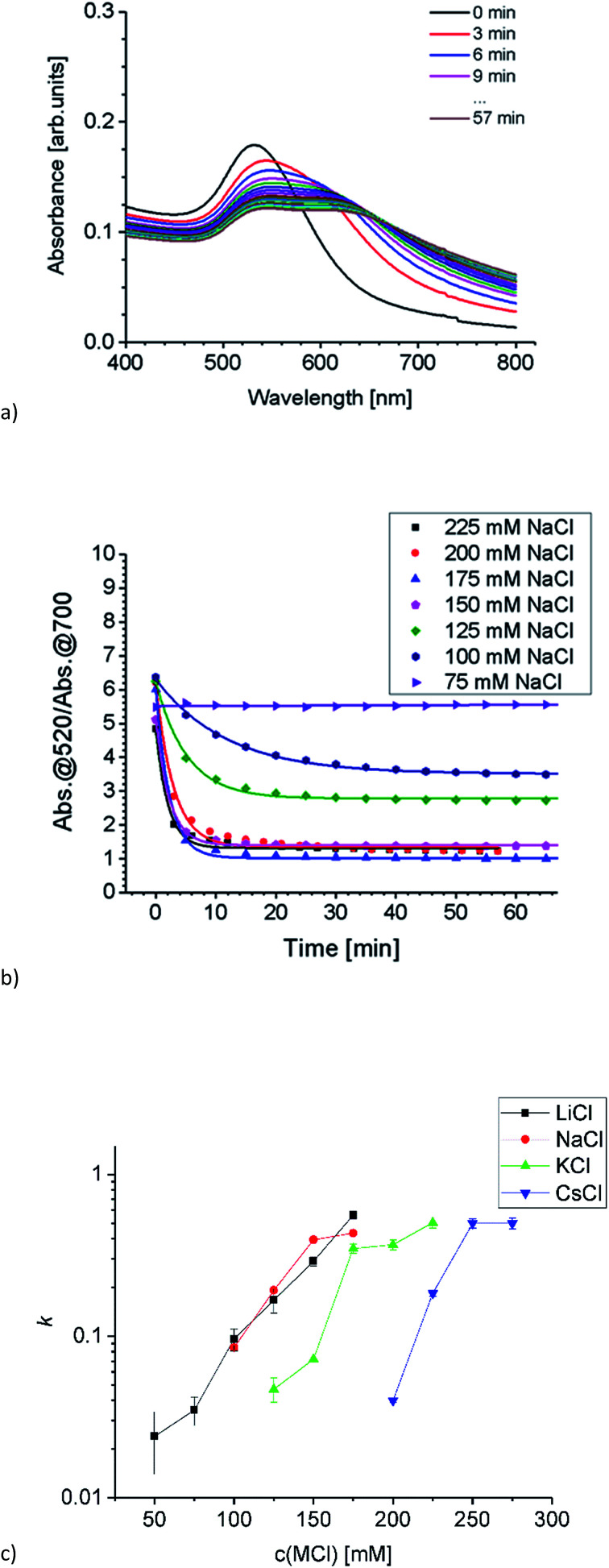
(a) Time dependent absorption spectra of Au-AOT in H_2_O/HCl at pH 3 after adding NaCl (final concentration 200 mM), spectra were taken every 3 minutes over 1 h. (b) Time course of the ratio *R* of absorbance for NaCl concentrations from 75 to 225 mM (symbols) and the respective fit curves (lines) fitted by a first order exponential decay function. (c) Semi-logarithmic plot of *k* as a function of electrolyte concentration for the time dependent aggregation of Au-AOT. The higher the value *k*, the faster the aggregation proceeds.

Aggregation of Au-AOT was investigated in the same manner as described for Au-MOA. For these positively charged AuNP we expect a less pronounced influence of the different cations, because ion pairing of the cationic –NH_3_^+^ end group with chloride anions takes place in the same degree for all investigated salts. Nevertheless, we expected a certain effect due to adsorption of cations to the neutral NH_2_-groups, which results in an increase of positive charge and therefore in a higher stability in highly concentrated salt solutions. The SEM images of the Au-AOT samples, which were prepared during the aggregation process, show fast formation of rather ellipsoidal aggregates with a size of *ca.* 50 nm after five minutes and of larger sized (200–300 nm) assemblies after 15 minutes (see Table S1 in the ESI†). In the UV-Vis, differing from the aggregation behaviour of Au-MOA, where the aggregation leads to formation of a new absorption maximum at higher wavelength, the respective spectra of the time dependent measurements for Au-AOT show an increase of the absorption within the wavelength range from 600 nm to 800 nm, but no distinct new maximum (Fig. 6a). Therefore, the ratio *R* of absorbance was determined as the ratio of the absorption at 520 nm to the absorption at 700 nm (Fig. 6b). Fitting of the time dependent ratio *R* of absorbance retrieved the aggregation constant *k* for the aggregation of Au-AOT with MCl (Li, Na, K, Cs) at salt concentrations from 100–275 mM (see Fig. S32–S56 in the ESI† for all corresponding UV-Vis spectra).

The evaluation of the *k* values shows that aggregation occurs at low salt concentrations for LiCl and NaCl while, in the case of KCl aggregation starts at higher salt concentration, and we observed highest stability with CsCl (Fig. 6c). These observations are in good accordance with the trend expected from literature.^2,7,12^ The kosmotropic cations Li^+^ and Na^+^ do not adsorb to the neutral chaotropic amine groups of Au-AOT, therefore no additional stabilisation due to added positive charges is possible. Accordingly, aggregation is observed at low salt concentrations with kosmotropic cations, *e.g.* at 125 mM with LiCl. The chaotropic cations K^+^ and Cs^+^ adsorb to the amine end groups and thereby enhance the positive charge of the Au-AOT which results in a higher stability of these AuNP against salt induced aggregation, *e.g.* Au-AOT are still stable in 200 mM CsCl as electrolyte. Hence, the aggregation behavior of Au-AOT in different monovalent chloride salts follows the trend expected from the theory of ion specific effects.^2,7,12^

How do these results correlate to the adsorption of the investigated AuNP on AuPd or Pt metal surfaces? We proposed that the binding of the AuNP is achieved upon coordinative binding of available electron pairs of the functional end groups –COO^−^ and –NH_2_ (Fig. 1b). Therefore, in case of Au-AOT, the covering density should decrease with increasing adsorption of cations to the NH_2_-groups, *i.e.* with increasing ion radius of M^+^. From the aggregation experiments with Au-MOA we can conclude that the covering density should increase with increasing ion radius, with an exception for Li^+^. A comparison with the experimentally obtained covering densities (see Fig. 3 and 4) confirms these expectations, which proves the proposed coordinative binding mechanism through the AuNPs' terminal groups.

## Conclusions

This experimental study gives evidence of ion specific effects on the adsorption of thiol stabilised AuNP with carboxylic acid (Au-MOA) and amine (Au-AOT) terminal groups, respectively, on platinum and gold/palladium surfaces. We investigated the covering density of Au-MOA and Au-AOT on platinum and gold/palladium as well as their salt induced precipitation as function of different monovalent salt solutions (MCl, M^+^ = Li^+^, Na^+^, K^+^, Cs^+^). We found for Au-MOA the highest covering density upon addition of Li^+^, a minimum with Na^+^ and in between for K^+^ and Cs^+^. The aggregation experiments led to comparable trends, *i.e.*, for Au-MOA fast aggregation with Na^+^ and K^+^, delayed aggregation with Li^+^ and longest stability in Cs^+^. In the adsorption experiments with Au-AOT a maximum covering density was observed with Li^+^, which decreased with decreasing ion radius of the added cation. The salt induced aggregation of Au-AOT exhibited increasing stability in the order LiCl < NaCl < KCl < CsCl. The observed trends point to an increased stabilisation in solution with increasing ion radius of the added cations due to their adsorption to neutral amine groups. Based on a proposed binding of the AuNPs *via* donation of free electron pairs of the terminal groups to the metal surface, the results of the aggregation experiments fit well to the results of the adsorption experiments, which confirms our binding model.

To sum up, we could elucidate the interactions of the terminal groups –COO^−^ and –NH_2_ with different monovalent cations by combination of aggregation and adsorption experiments. These results allow further control of the self-assembly of charged NPs in solution and on metal surfaces with respect to covering density and 3D network formation, which can be utilized for the development of new sensors,^33^ metamaterials,^18^ and the implementation of functionalised AuNPs within nanoelectronic devices.^34^

## Conflicts of interest

There are no conflicts to declare.

## Supplementary Material

RA-008-C7RA10374C-s001
