# Supplementary material for: Ion specific effects on the immobilisation of charged gold nanoparticles on metal surfaces
Source: RSC Adv. 2018 Jan 5;8(3):1717–24. doi: 10.1039/c7ra10374c (PMC9077124; doi:10.1039/c7ra10374c)
Supplement: RA-008-C7RA10374C-s001 [file RA-008-C7RA10374C-s001.pdf]

Electronic Supplementary Material (ESI) for RSC Advances.
This journal is © The Royal Society of Chemistry 2017

Electronic Supplementary Information

for

Ion Specific Effects on the Immobilisation of Charged Gold Nanoparticles on Metal Surfaces

C. Kaulen and U. Simon

1. JARA – FIT, RWTH Aachen University, 52074 Aachen, Germany and 2. Institute of Inorganic Chemistry, RWTH Aachen University, 52074 Aachen, Germany

1. Characterisation of Au-MOA

   a. UV-Vis

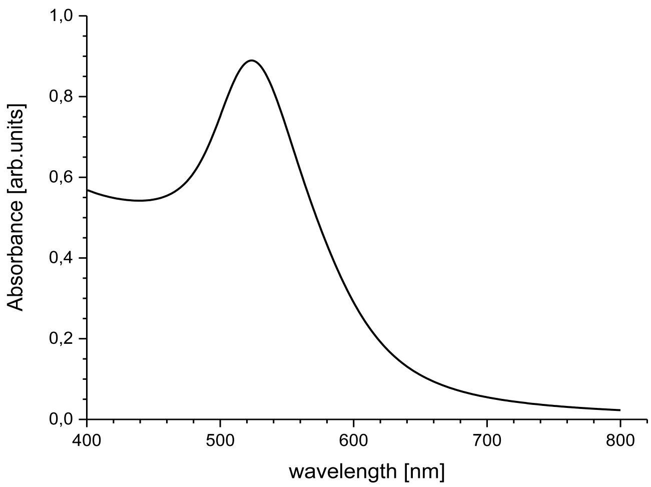

Figure S1: UV-vis absorbance spectrum of Au-MOA in ultrapure water

   b. DLS

Z.Average: 25.90 ±0.03 nm PDI: 0.26

Zeta-potential: -42.5 ± 9.8 mV in HEPES/TRIS buffer at pH 9

SEM-T

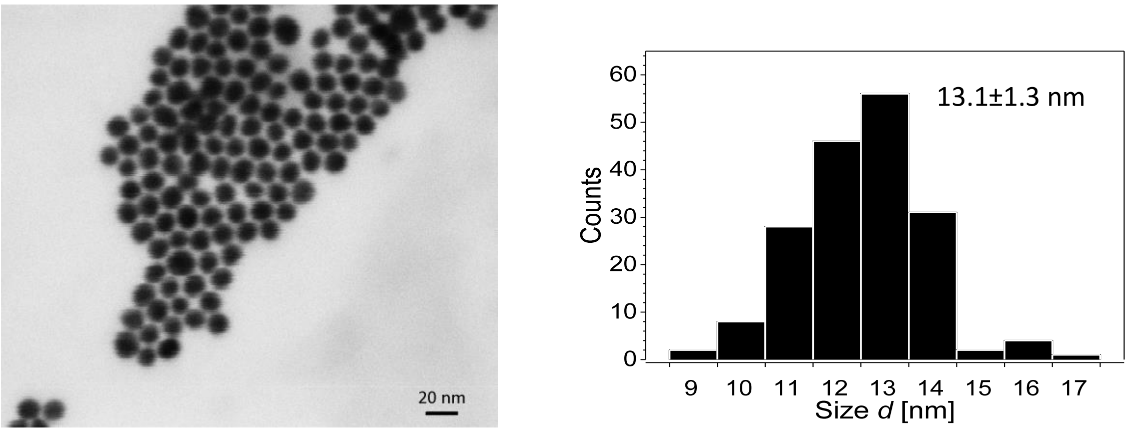

Mean diameter of Au-MOA: 13.1±1.3 nm

Figure S2: Representative SEM-T picture of Au-MOA and corresponding size distribution histogram

2. Characterisation of Au-AOT:

a. UV-Vis

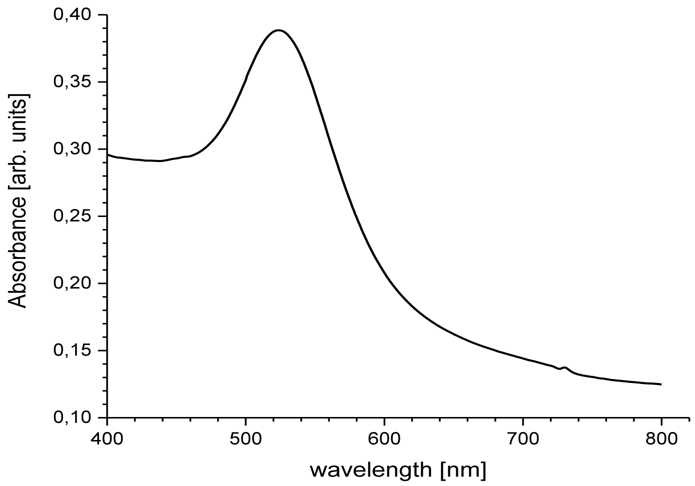

Figure S3: UV-vis absorbance spectrum of Au-AOT in ultrapure water

b. DLS

Z.Average: 38.04±0.50 PDI: 0.62±0.13

Zeta-potential: +60.7 ± 8.5 mV in diluted HCl at pH 3

c. SEM-T

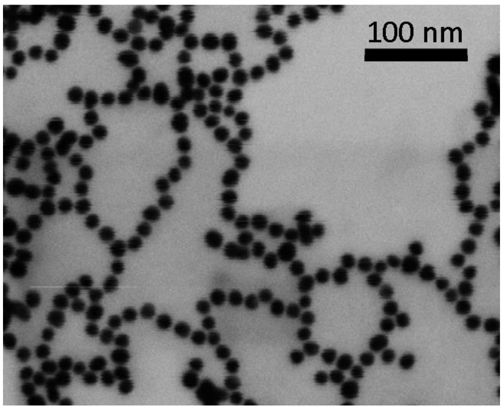

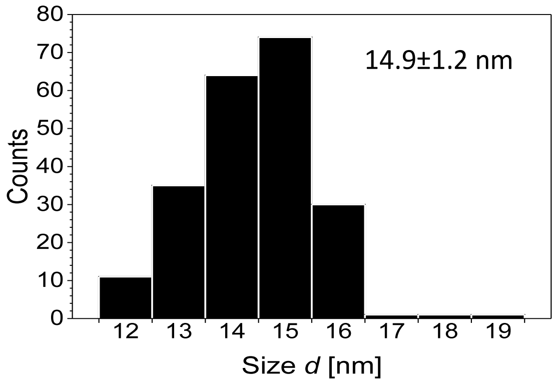

Mean diameter Au-AOT: 14.9±1.2 nm

Figure S4: Representative SEM-T picture of Au-AOT and corresponding size distribution histogram

3. Additional SEM images of the adsorption of Au-MOA on Pt/AuPd-substrates:

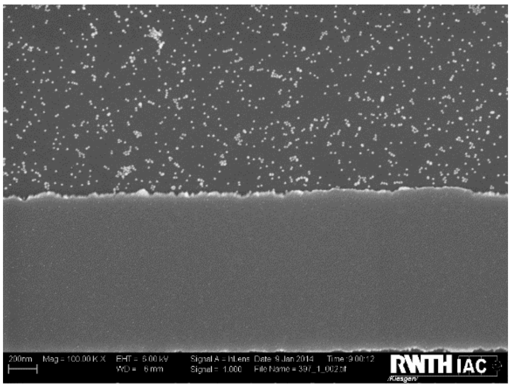

LiCl

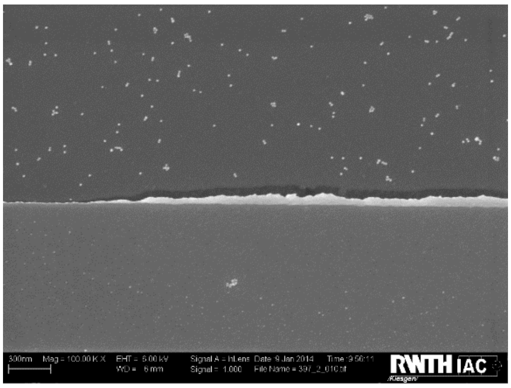

NaCl

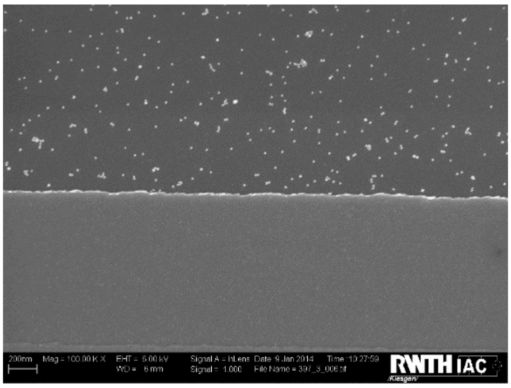

KCl

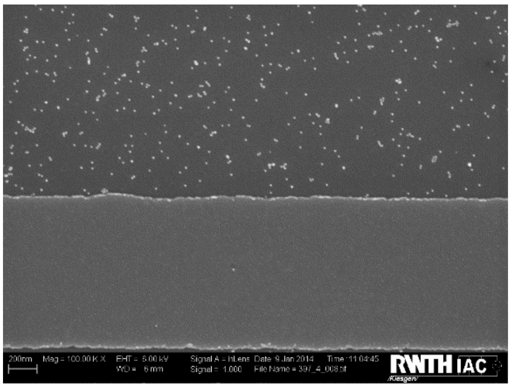

CsCl

Figure S5: Additional SEM images of adsorption experiments of Au-MOA with added monovalent salts MCl (M = Li, Na, K, Cs)

4. Additional SEM images of the adsorption of Au-AOT on Pt/AuPd-substrates:

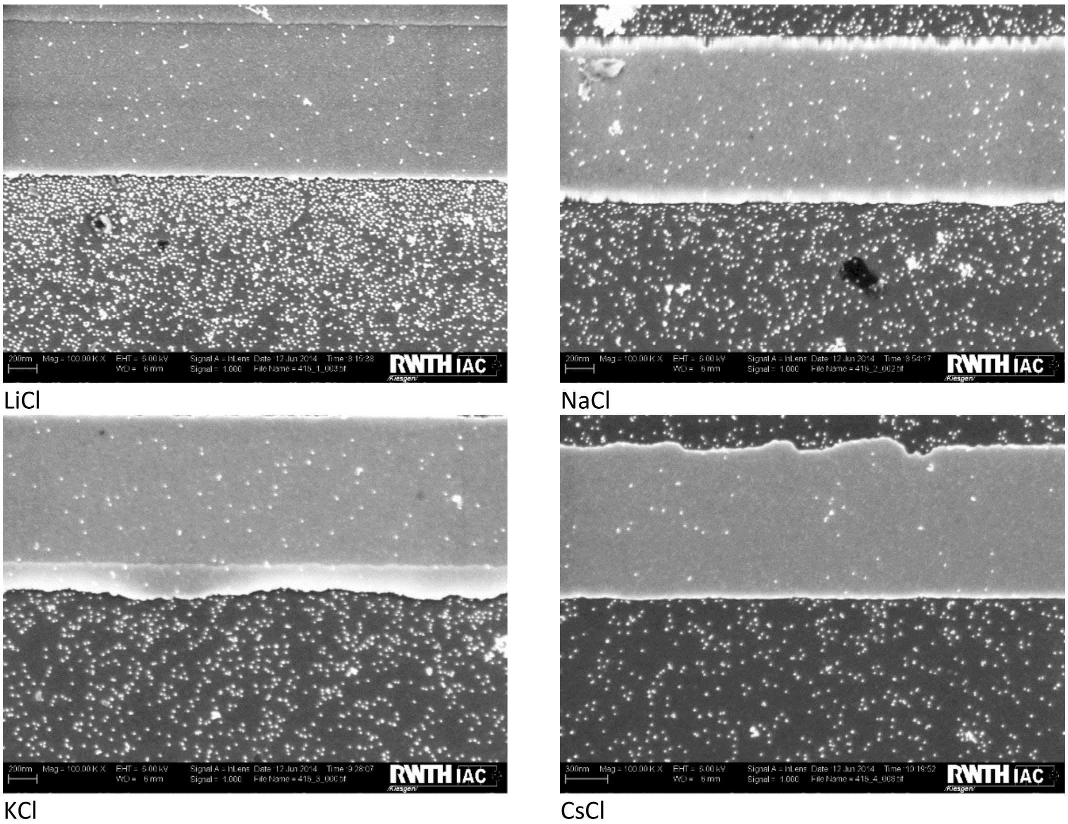

Figure S6: Additional SEM images of adsorption experiments of Au-AOT with added monovalent salts MCl (M = Li, Na, K, Cs)

5. SEM images of the formed aggregates

time	Au-MOA	Au-AOT
0	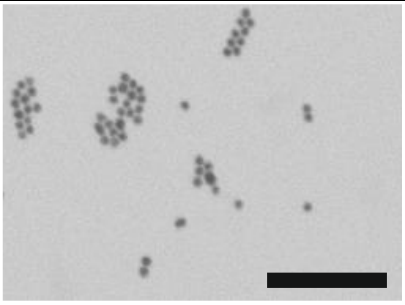	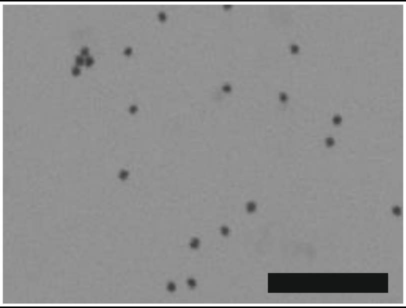
5	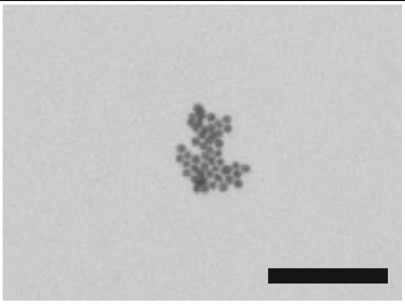	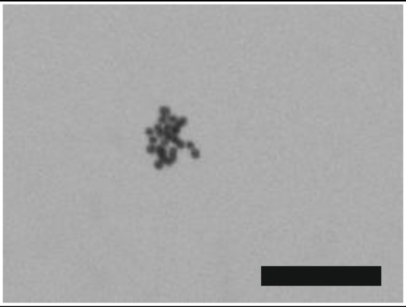
10	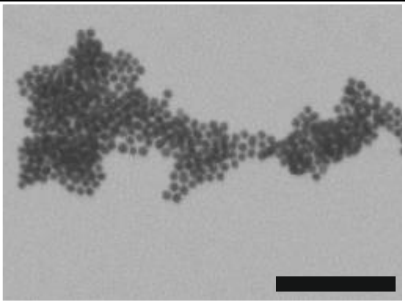	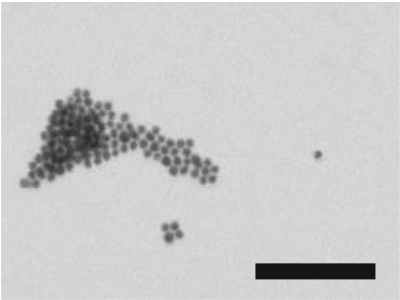
15	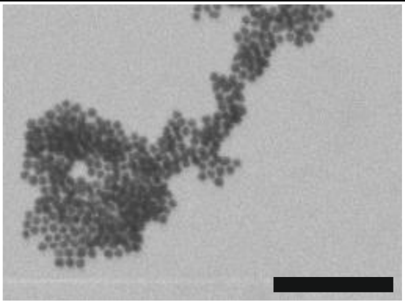	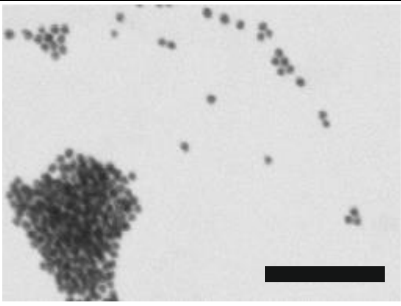
20	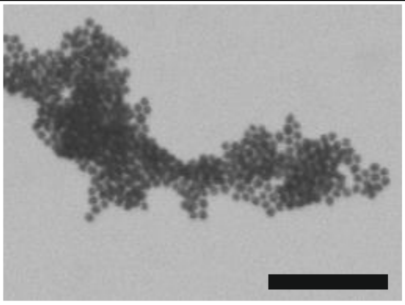	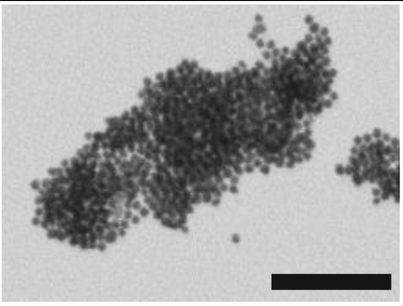

25	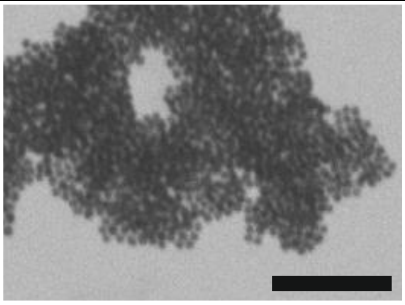	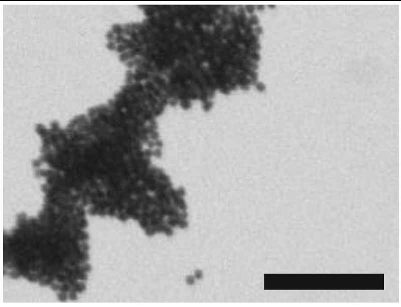
10µl samples of Au-MOA in 45 mmol NaCl and of Au-AOT in 125 mmol NaCl were taken every five minutes after mixing and deposited on the carbon surface of a TEM grid. After five additional minutes the solution was removed with a KIM wipe and the grids were allowed to dry at 20% air humidity and 20°C. SEM images were aquired with a high resolution Field Emission Scanning Electron Microscope in transmission mode (FE-SEM, LEO/ZEISS Supra 35 VP, Oberkochen, Germany). Scale bar represents 200 nm.		

6. Salt Induced Aggregation of Au-MOA with LiCl

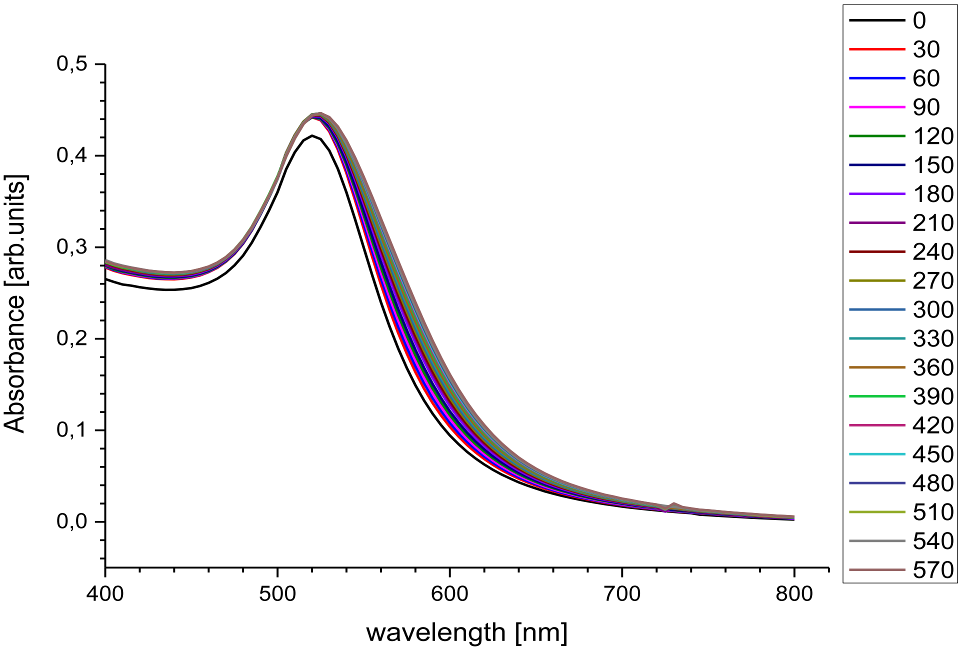

Figure S7: Time dependent absorption spectra of Au-MOA in HEPES/TRIS at pH 9 and 50 mMol LiCl

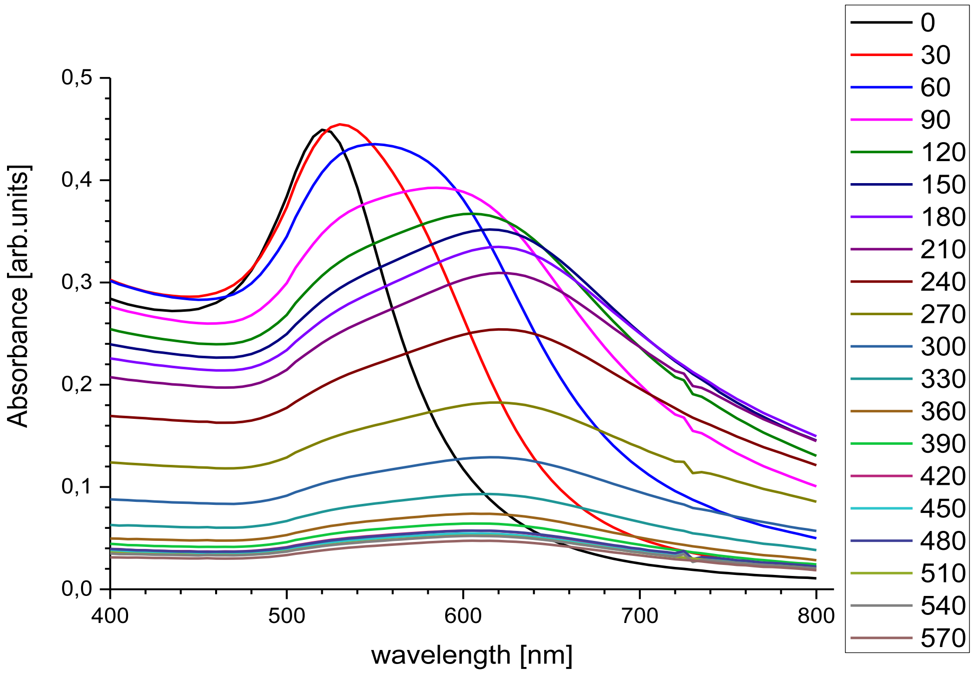

Figure S8: Time dependent absorption spectra of Au-MOA in HEPES/TRIS at pH 9 and 60 mMol LiCl

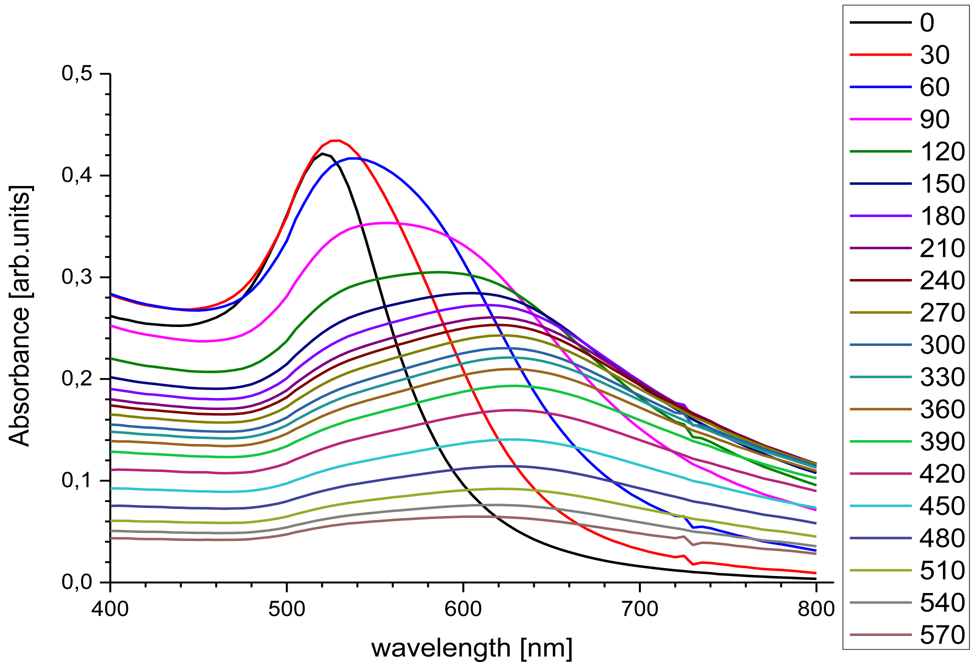

Figure S9: Time dependent absorption spectra of Au-MOA in HEPES/TRIS at pH 9 and 70 mMol LiCl

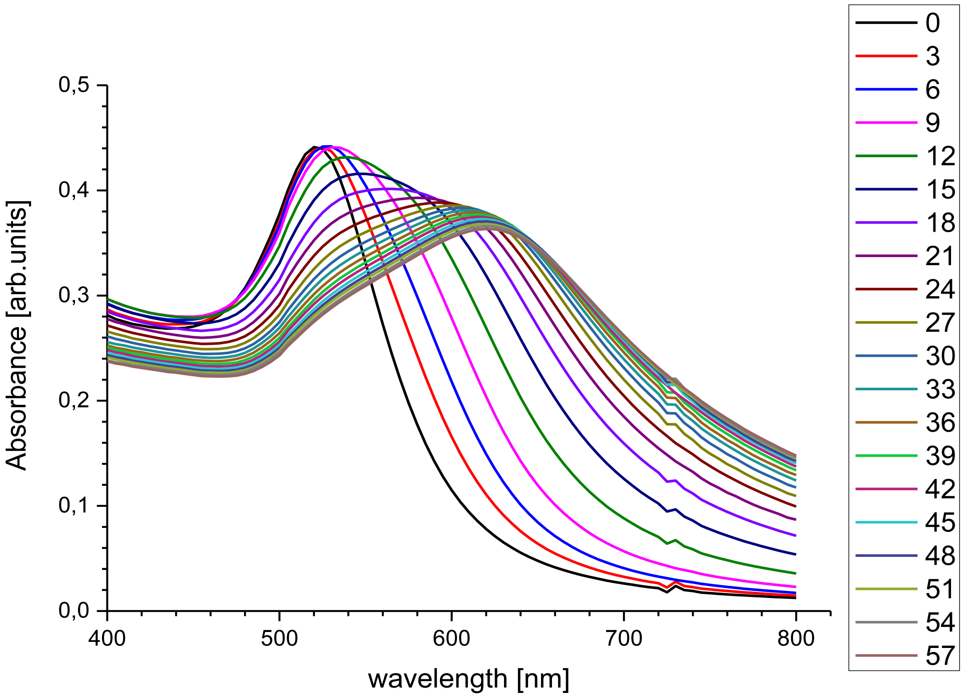

Figure S10: Time dependent absorption spectra of Au-MOA in HEPES/TRIS at pH 9 and 80 mMol LiCl

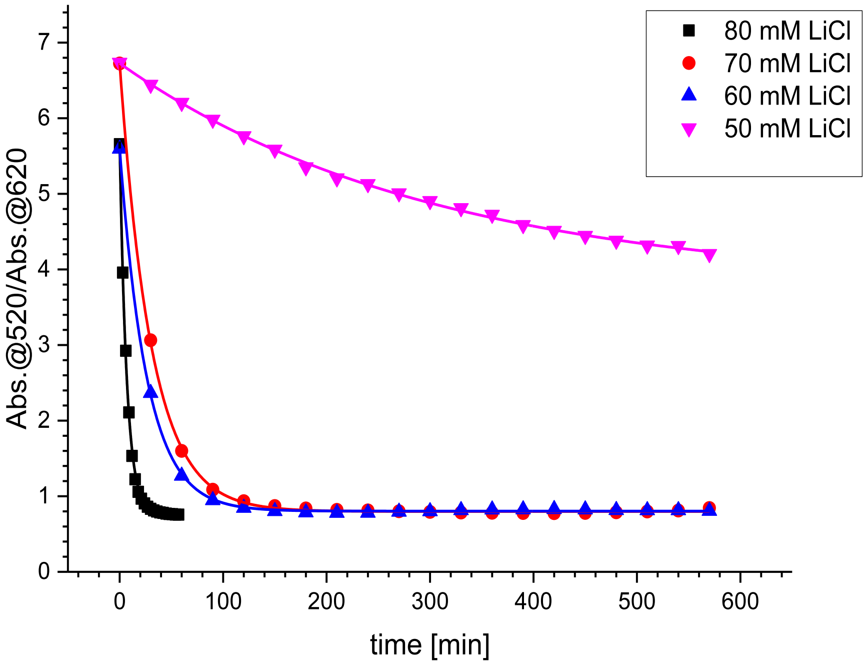

Figure S11: Progression of the ratio of absorbance *R* (symbols) for LiCl concentrations from 50 mM to 80 mM and the respective fit curves (lines) fitted by a first order exponential decay function.

7. Salt Induced Aggregation of Au-MOA with NaC

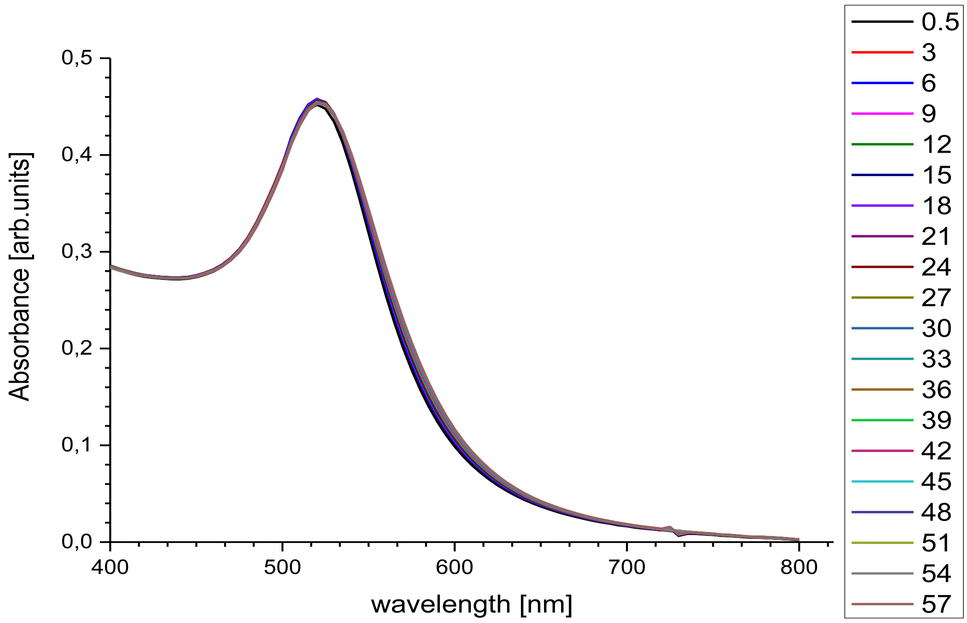

Figure S12: Time dependent absorption spectra of Au-MOA in HEPES/TRIS at pH 9 and 40 mMol NaCl

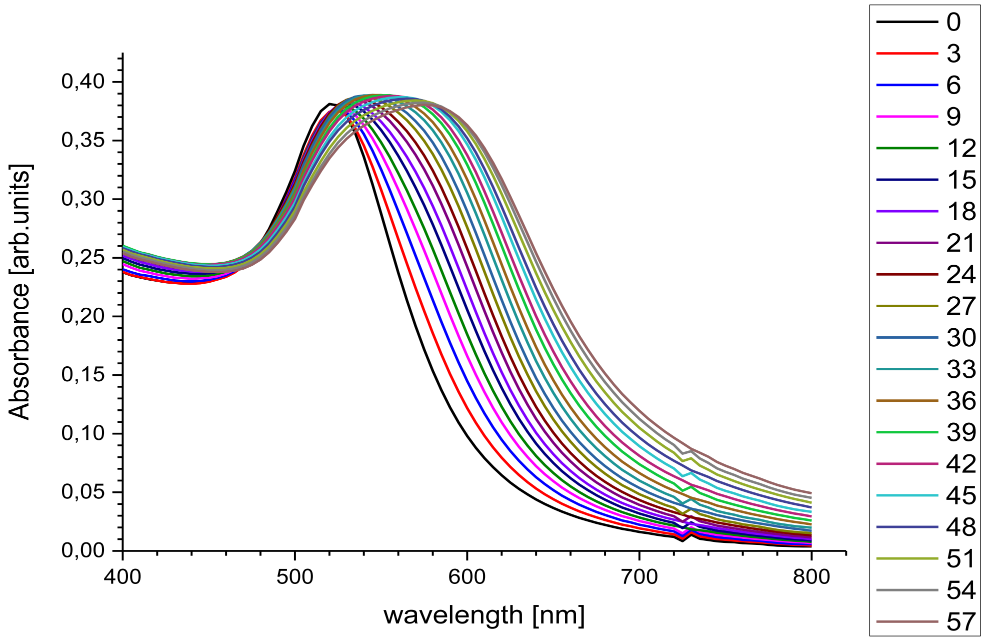

Figure S13: Time dependent absorption spectra of Au-MOA in HEPES/TRIS at pH 9 and 45 mMol NaCl

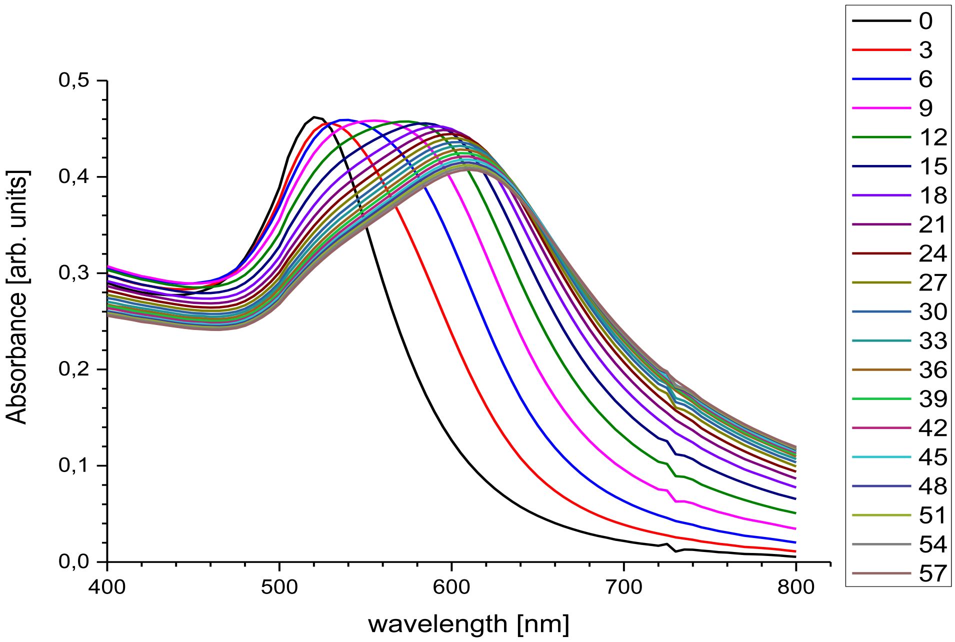

Figure S14: Time dependent absorption spectra of Au-MOA in HEPES/TRIS at pH 9 and 50 mMol NaCl

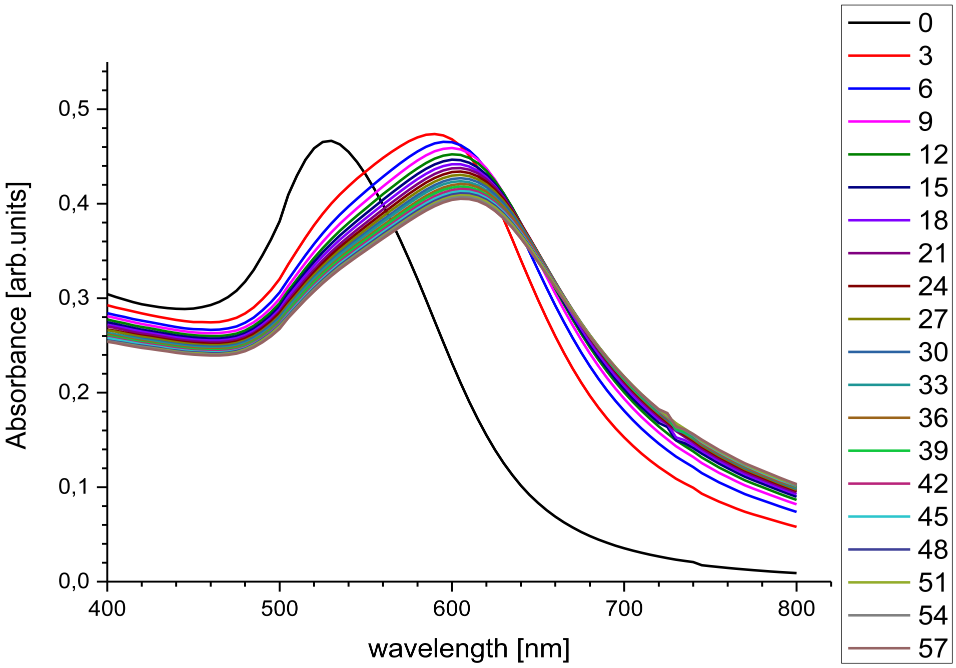

Figure S15: Time dependent absorption spectra of Au-MOA in HEPES/TRIS at pH 9 and 60 mMol NaCl

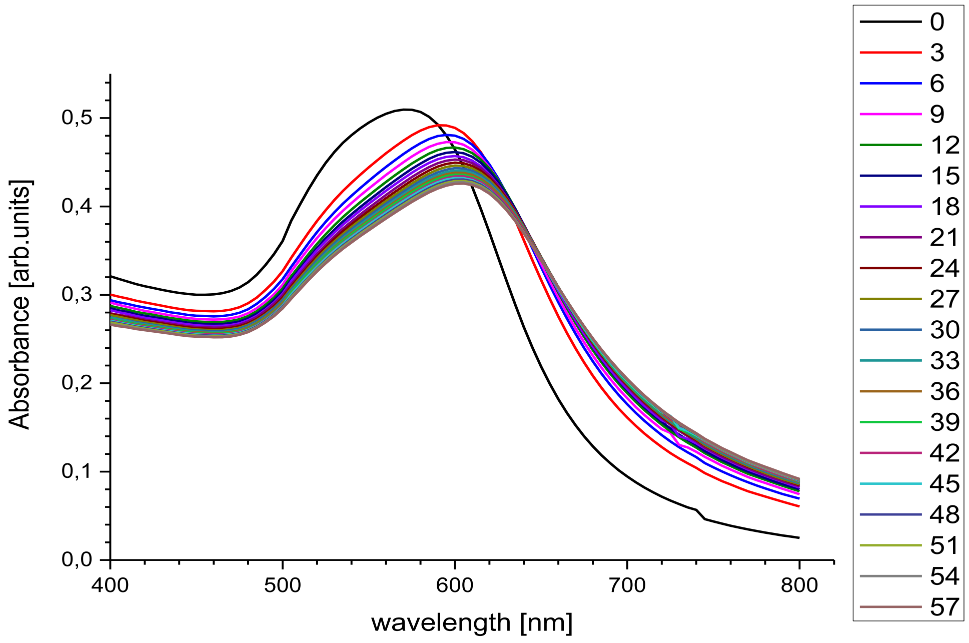

Figure S16: Time dependent absorption spectra of Au-MOA in HEPES/TRIS at pH 9 and 70 mMol NaCl

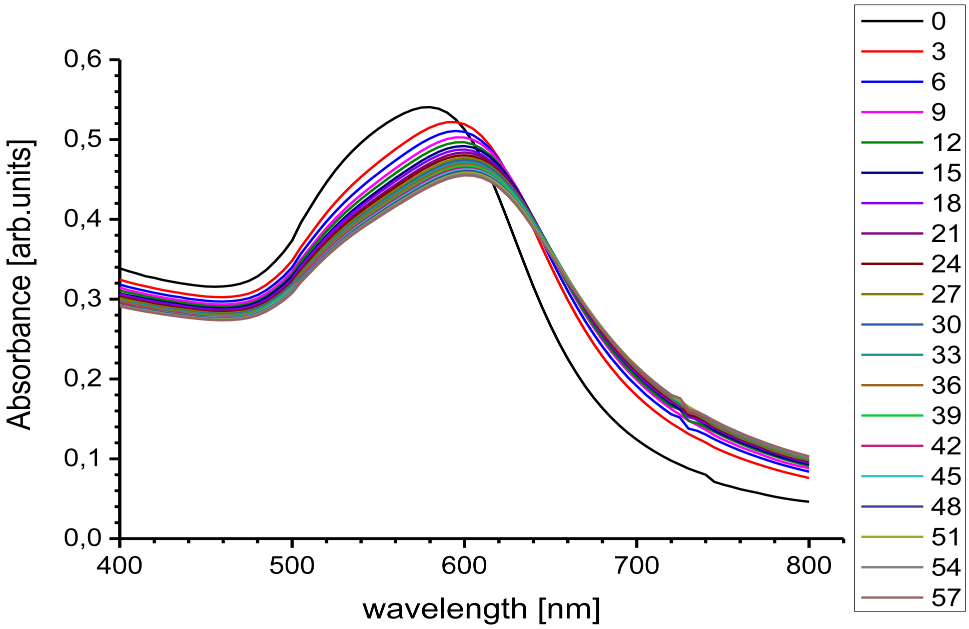

Figure S17: Time dependent absorption spectra of Au-MOA in HEPES/TRIS at pH 9 and 80 mMol NaCl

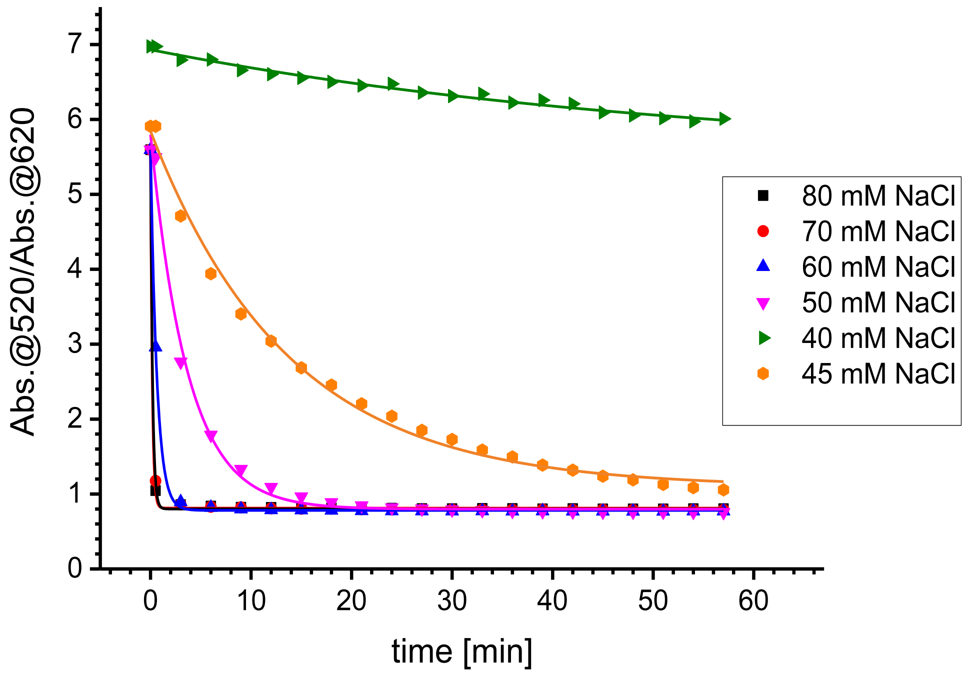

Figure S18: Progression of the ratio of absorbance *R* (symbols) for NaCl concentrations from 40 mM to 80 mM and the respective fit curves (lines) fitted by a first order exponential decay function.

8. Salt Induced Aggregation of Au-MOA with KCl

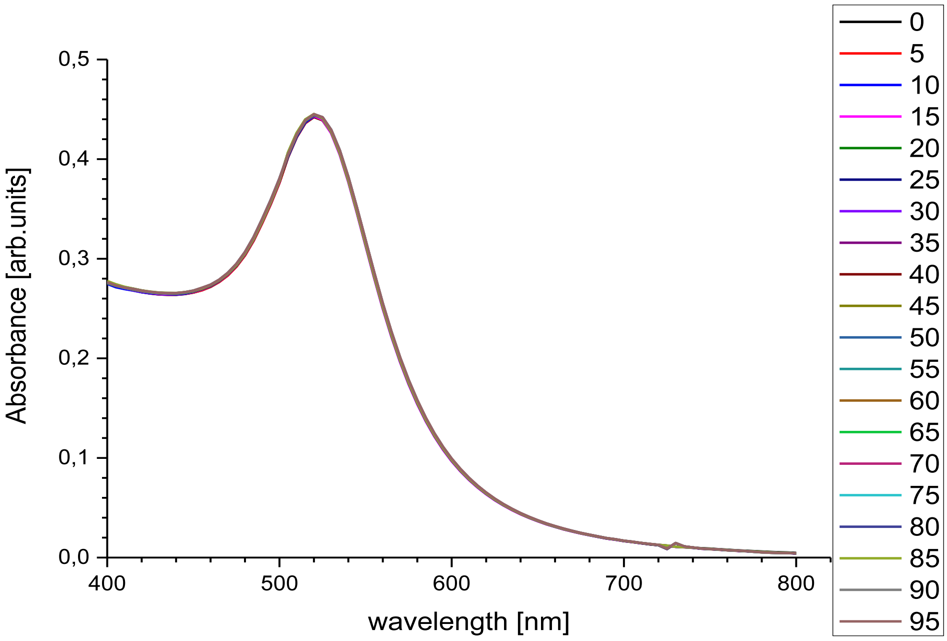

Figure S19: Time dependent absorption spectra of Au-MOA in HEPES/TRIS at pH 9 and 30 mMol KCl

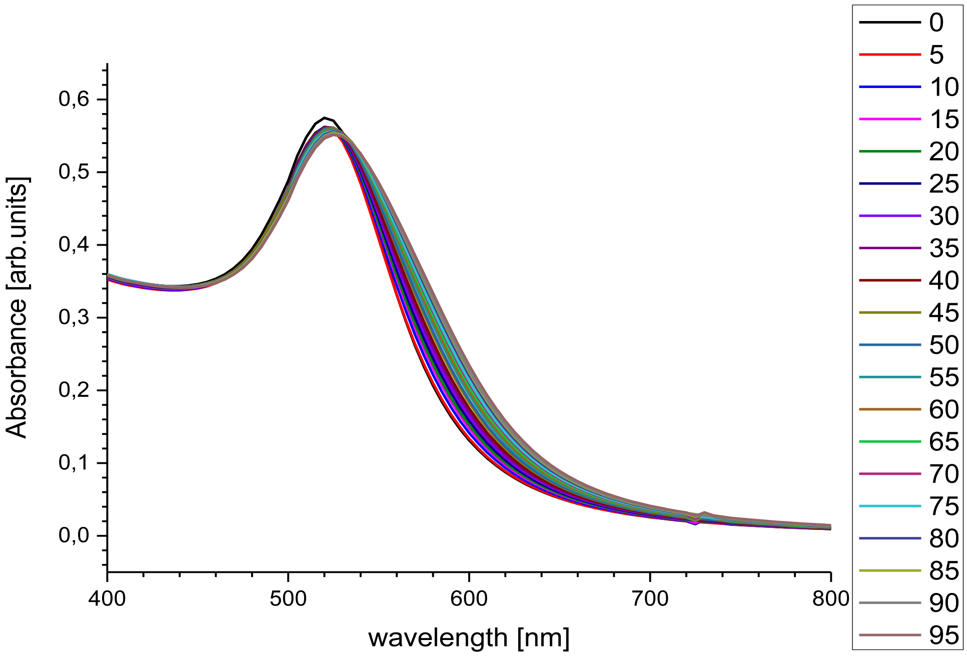

Figure S20: Time dependent absorption spectra of Au-MOA in HEPES/TRIS at pH 9 and 40 mMol KCl

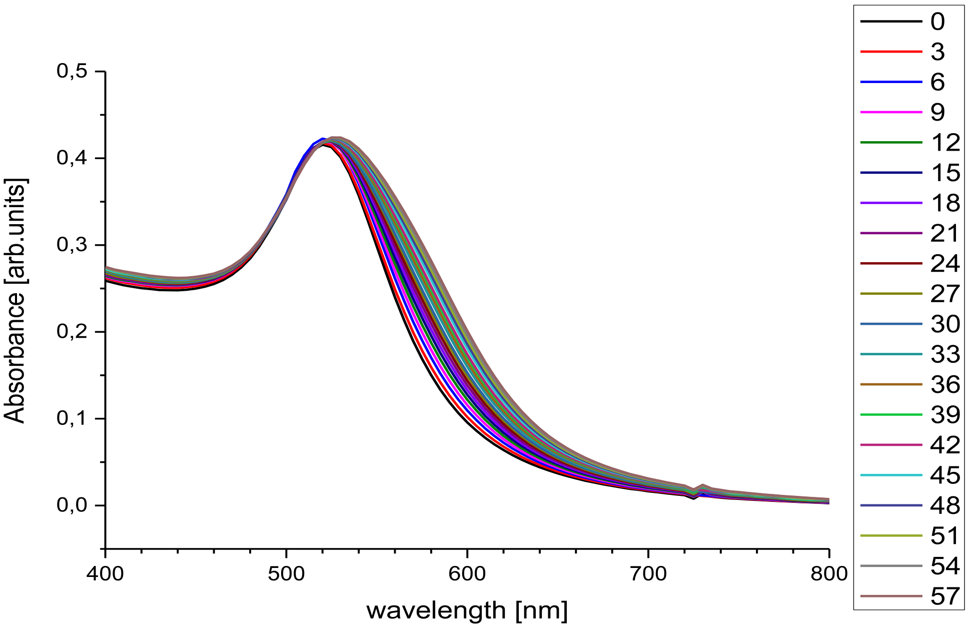

Figure S21: Time dependent absorption spectra of Au-MOA in HEPES/TRIS at pH 9 and 45 mMol KCl

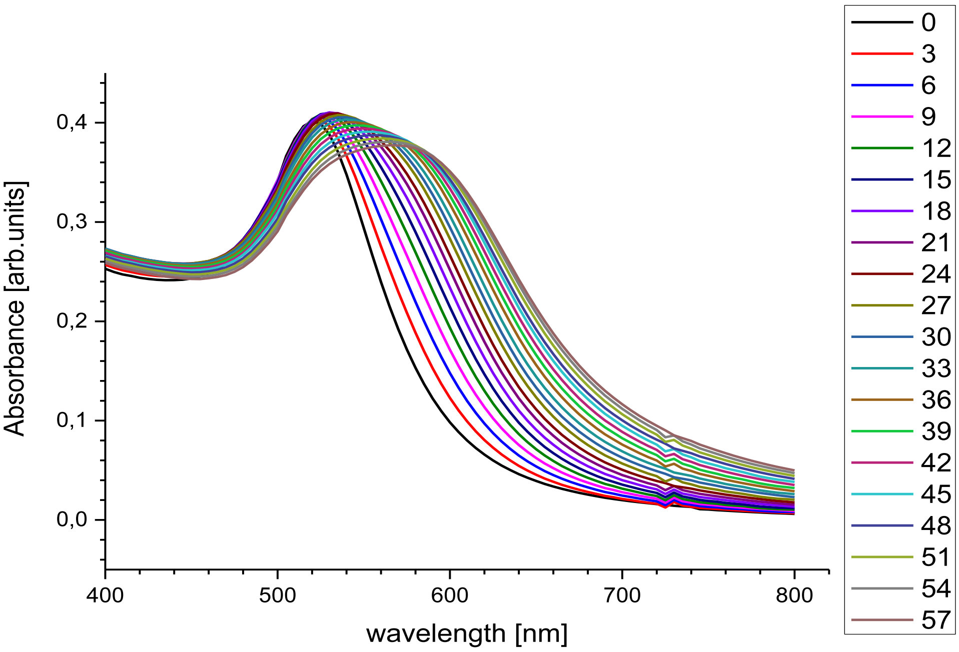

Figure S22: Time dependent absorption spectra of Au-MOA in HEPES/TRIS at pH 9 and 50 mMol KCl

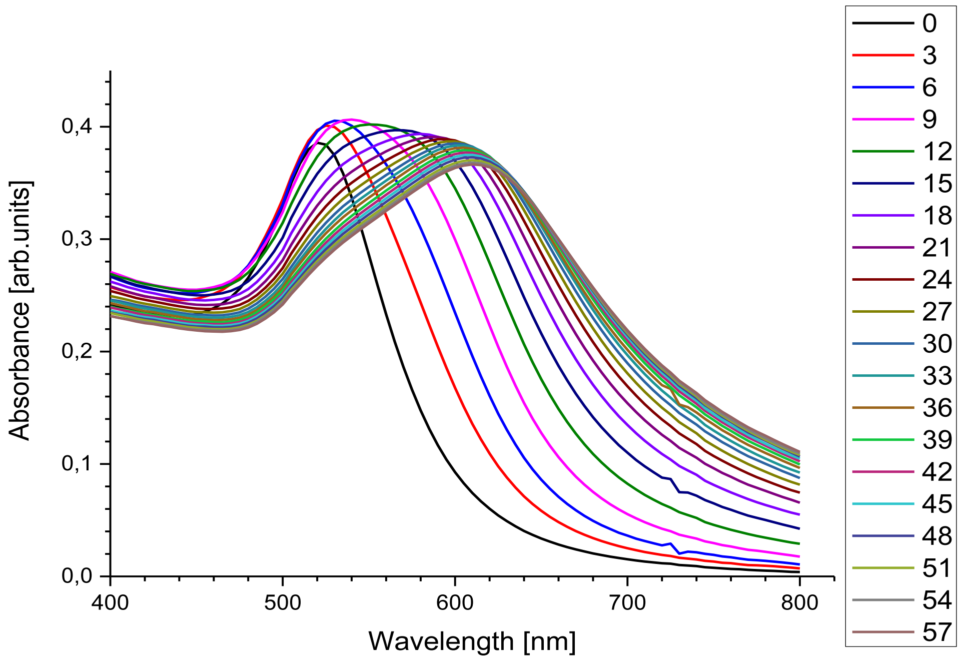

Figure S23: Time dependent absorption spectra of Au-MOA in HEPES/TRIS at pH 9 and 60 mMol KCl

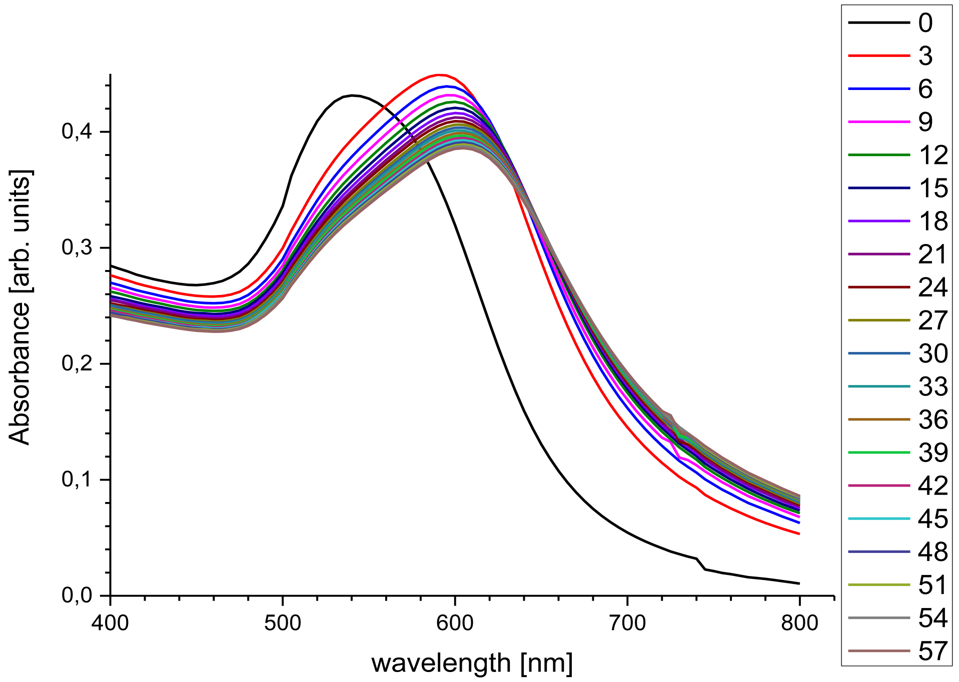

Figure S24: Time dependent absorption spectra of Au-MOA in HEPES/TRIS at pH 9 and 70 mMol KCl

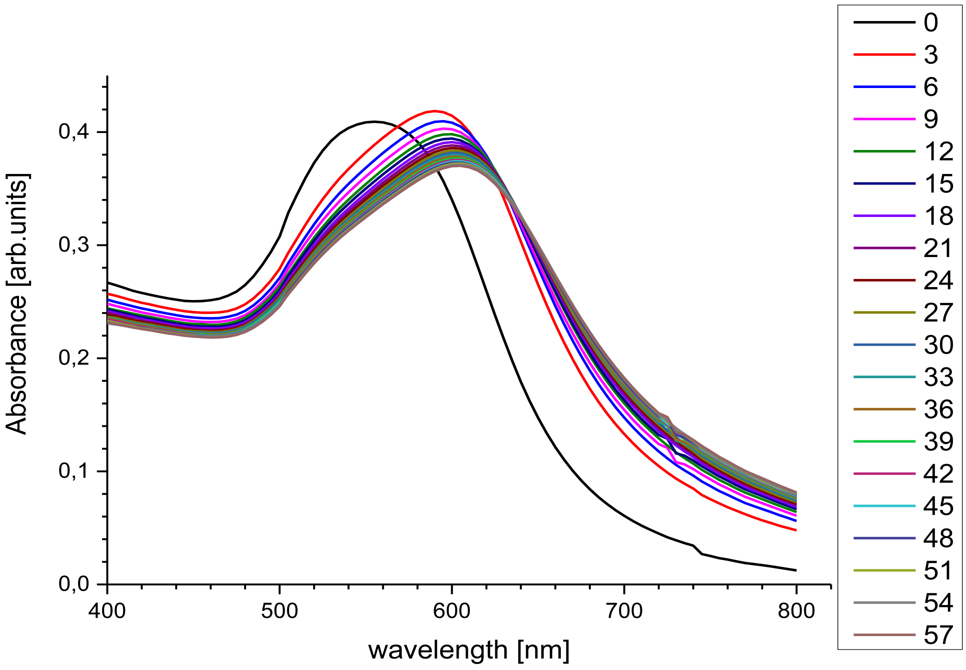

Figure S25: Time dependent absorption spectra of Au-MOA in HEPES/TRIS at pH 9 and 80 mMol KCl

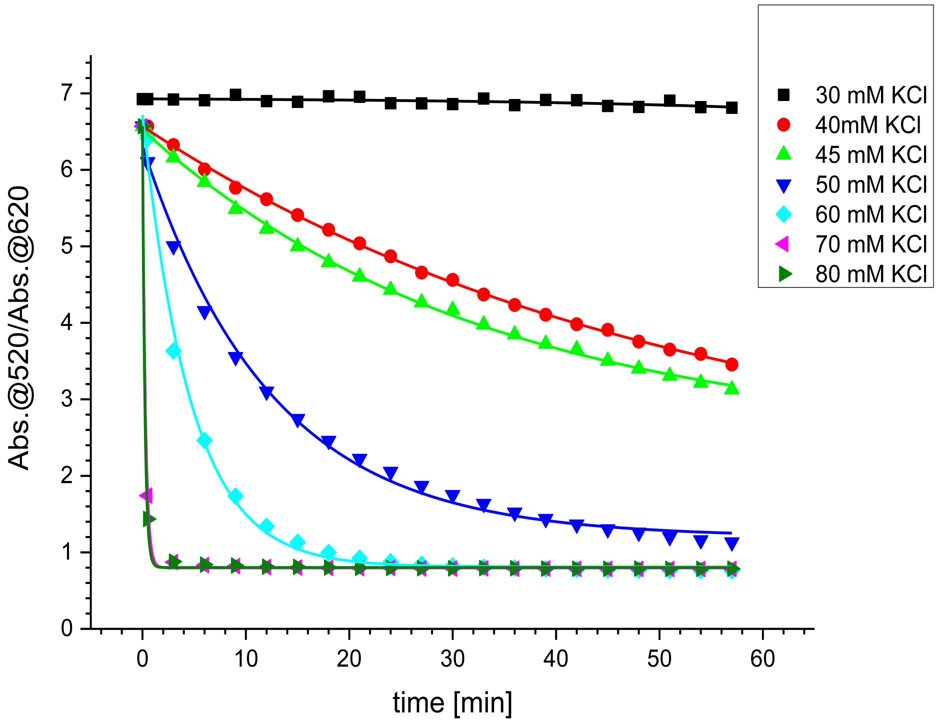

Figure S26: Progression of the ratio of absorbance *R* (symbols) for KCl concentrations from 30 mM to 80 mM and the respective fit curves (lines) fitted by a first order exponential decay function.

9. Salt Induced Aggregation of Au-MOA with CsCl

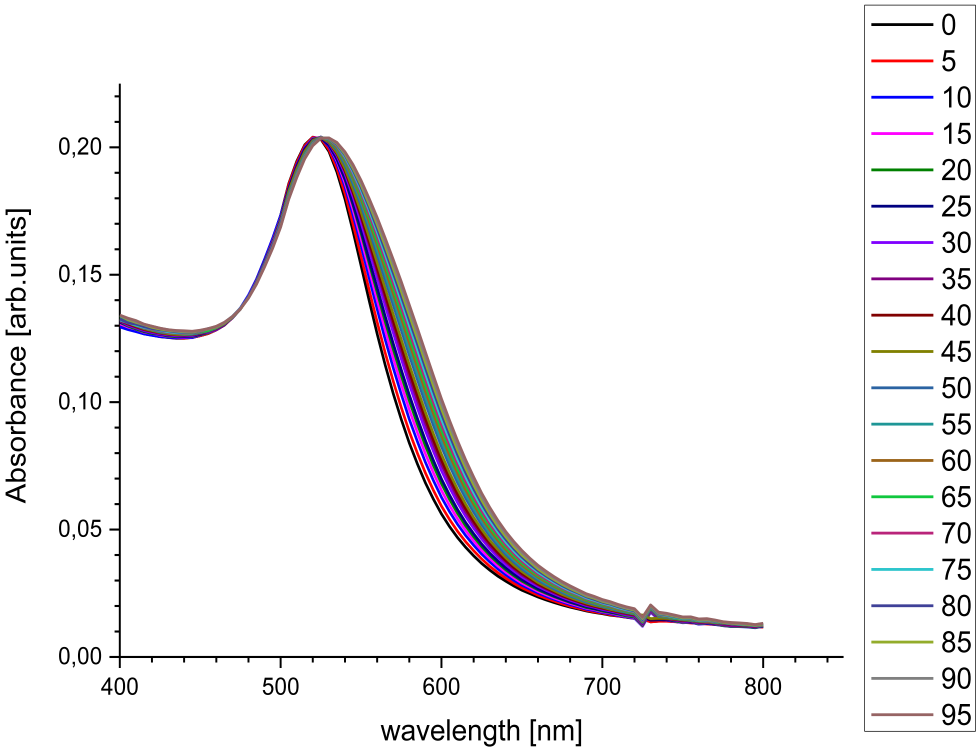

Figure S27: Time dependent absorption spectra of Au-MOA in HEPES/TRIS at pH 9 and 90 mMol CsCl

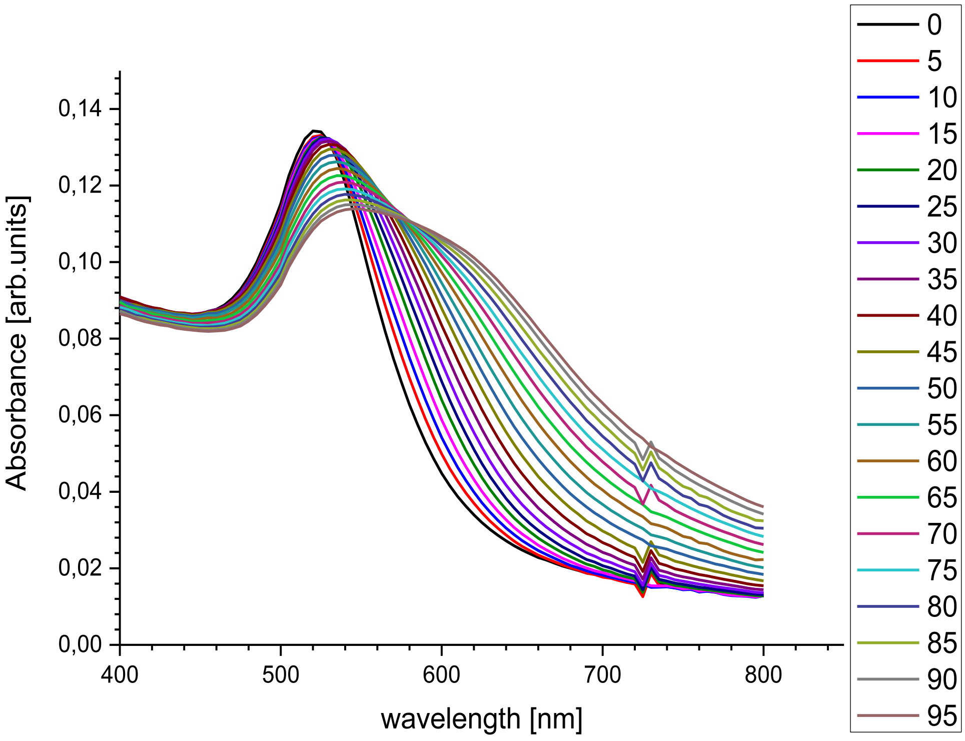

Figure S28: Time dependent absorption spectra of Au-MOA in HEPES/TRIS at pH 9 and 100 mMol CsCl

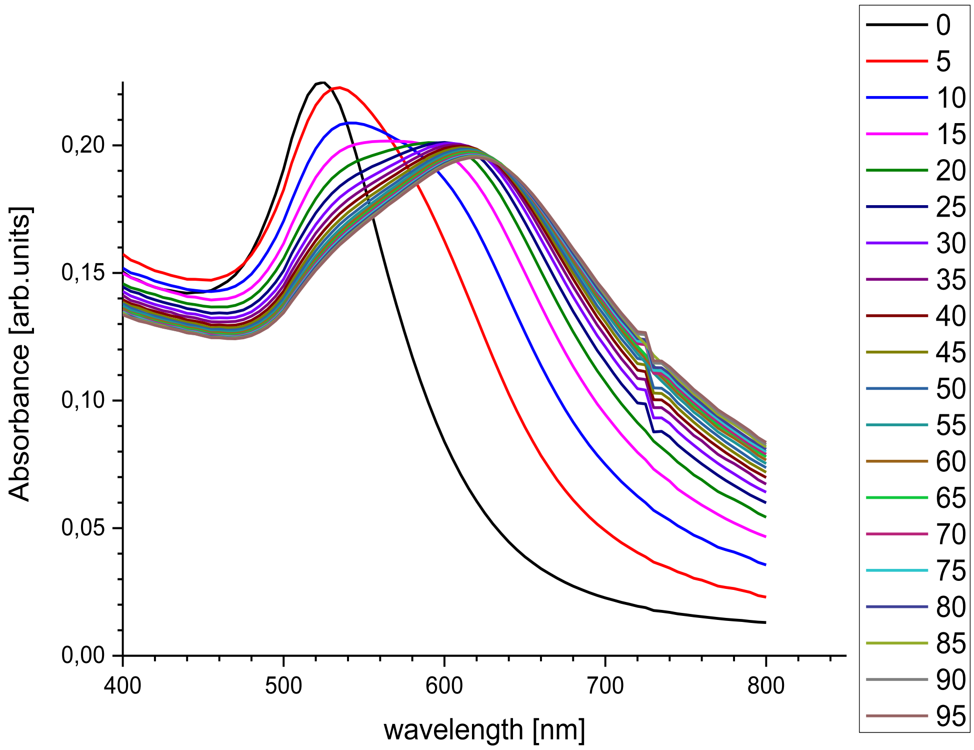

Figure S29: Time dependent absorption spectra of Au-MOA in HEPES/TRIS at pH 9 and 110 mMol CsCl

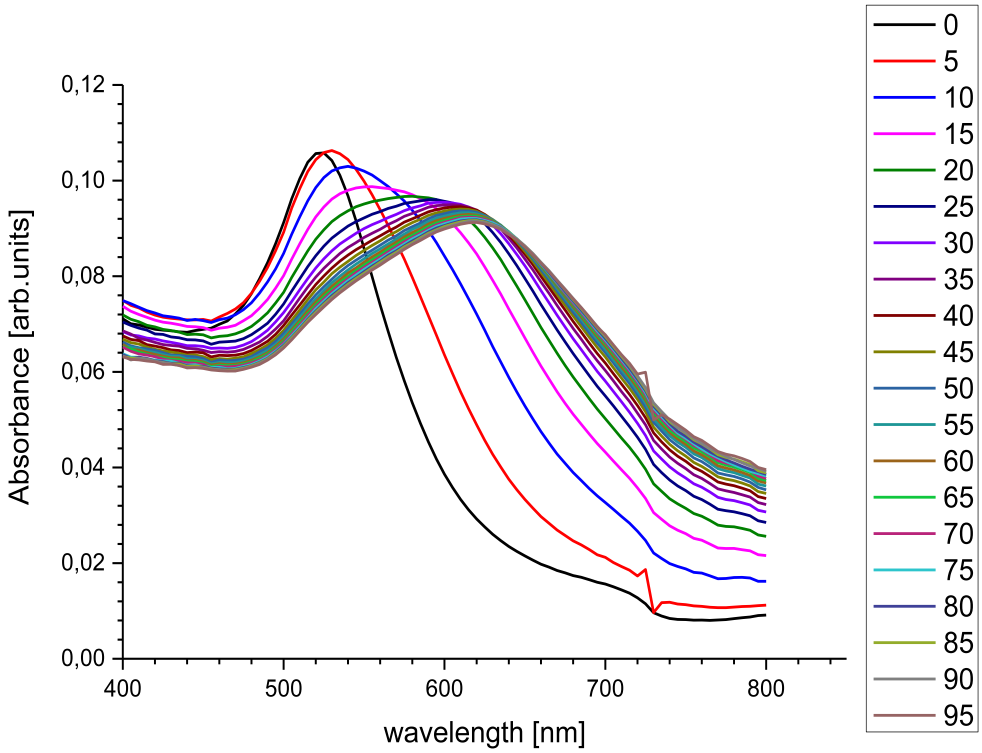

Figure S30: Time dependent absorption spectra of Au-MOA in HEPES/TRIS at pH 9 and 120 mMol CsCl

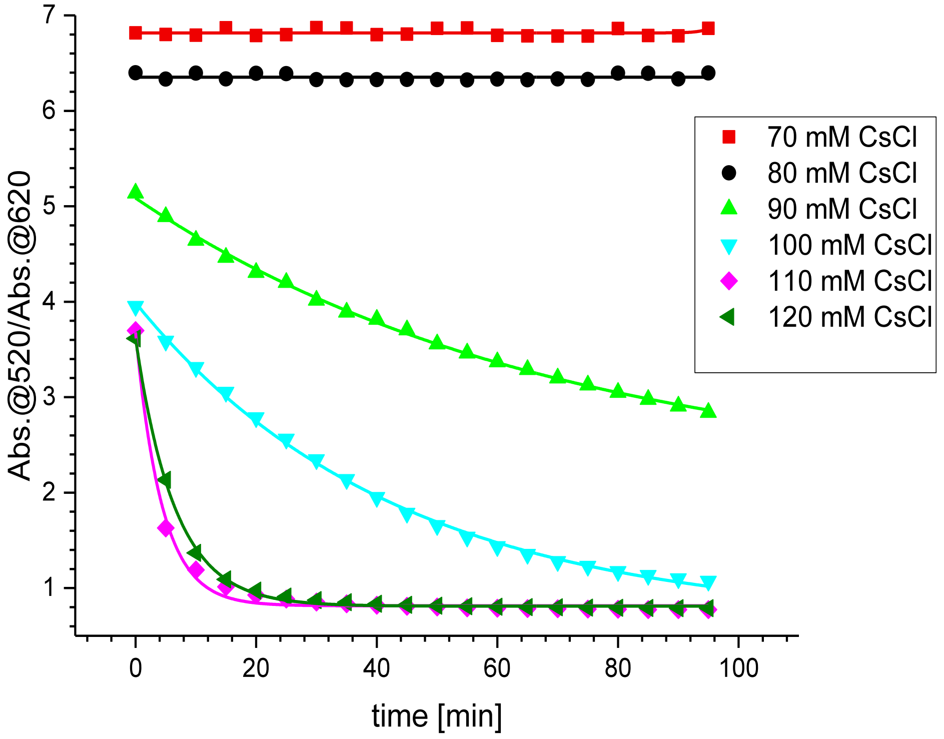

Figure S31: Progression of the ratio of absorbance *R* (symbols) for CsCl concentrations from 70 mM to 120 mM and the respective fit curves (lines) fitted by a first order exponential decay function.

10. Salt Induced Aggregation of Au-AOT with LiCl

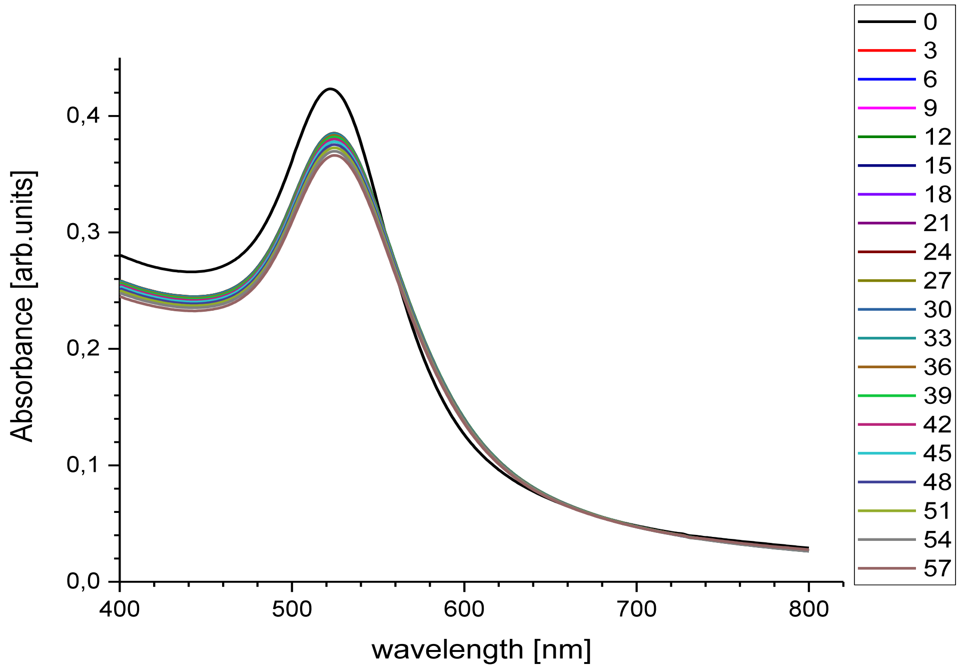

Figure S32: Time dependent absorption spectra of Au-AOT in diluted HCl at pH 3 and 100 mMol LiCl

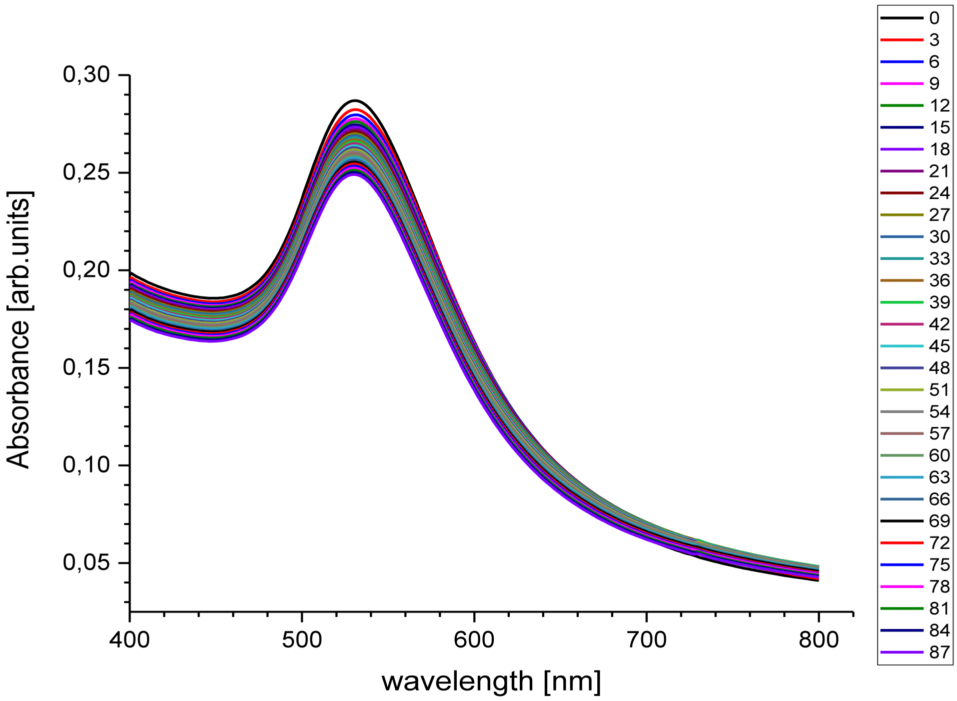

Figure S33: Time dependent absorption spectra of Au-AOT in diluted HCl at pH 3 and 125 mMol LiCl

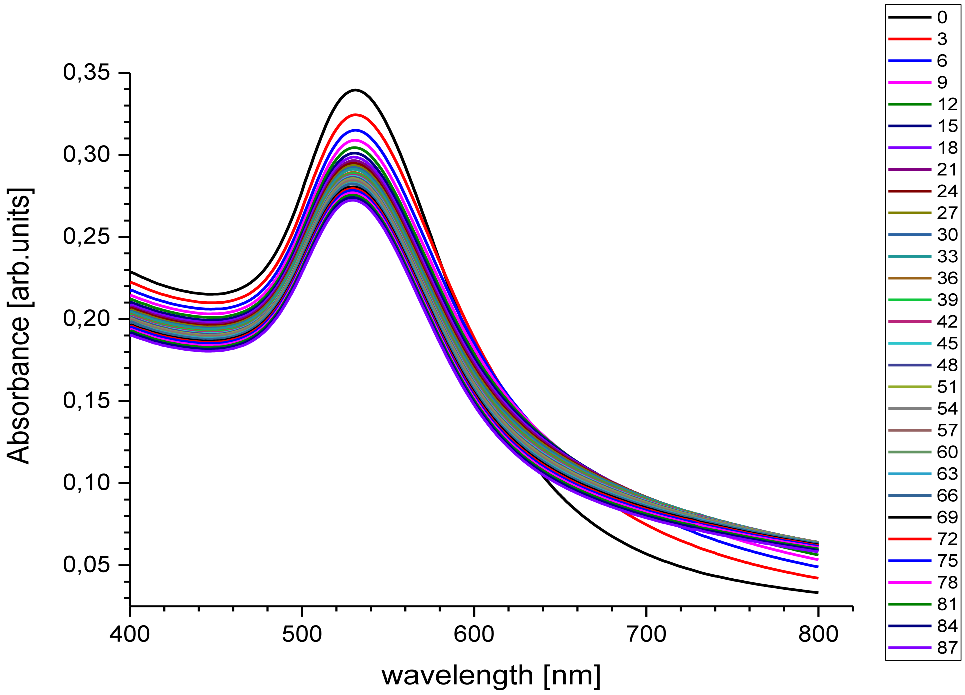

Figure S34: Time dependent absorption spectra of Au-AOT in diluted HCl at pH 3 and 150 mMol LiCl

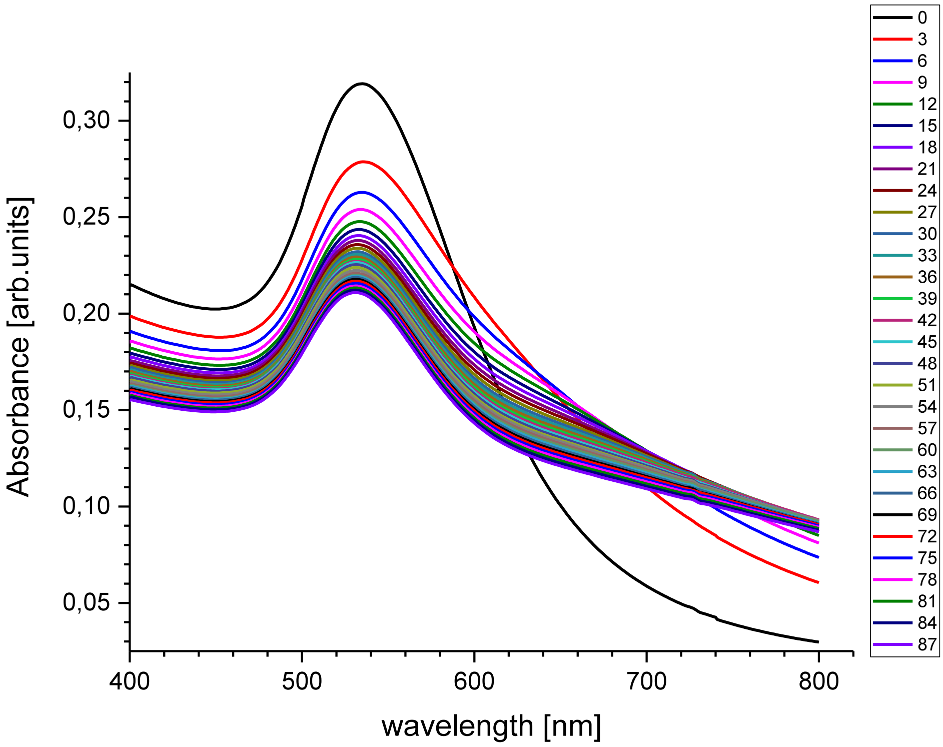

Figure S35: Time dependent absorption spectra of Au-AOT in diluted HCl at pH 3 and 175 mMol LiCl

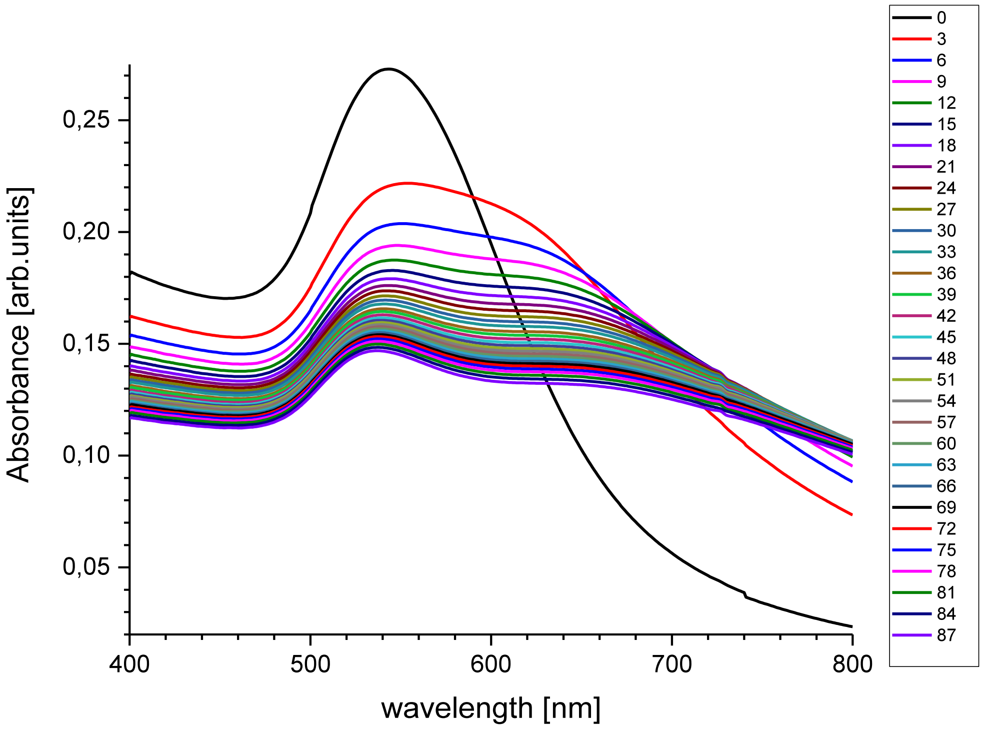

Figure S36: Time dependent absorption spectra of Au-AOT in diluted HCl at pH 3 and 200 mMol LiCl

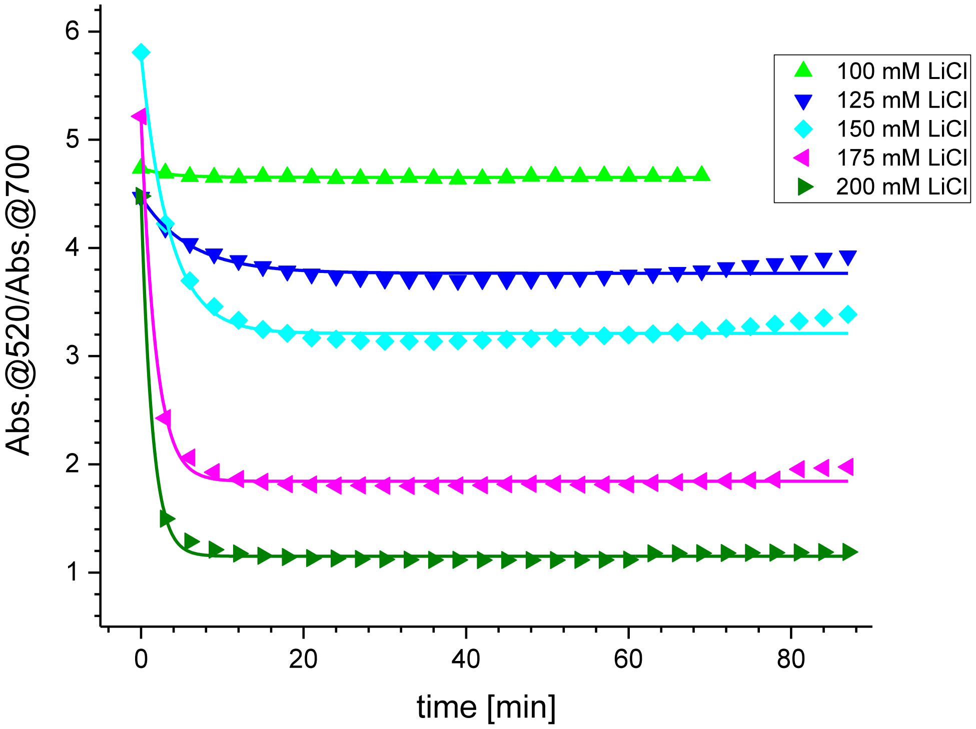

Figure S37: Progression of the ratio of absorbance *R* (symbols) for LiCl concentrations from 100 mM to 200 mM and the respective fit curves (lines) fitted by a first order exponential decay function.

11. Salt Induced Aggregation of Au-AOT with NaCl

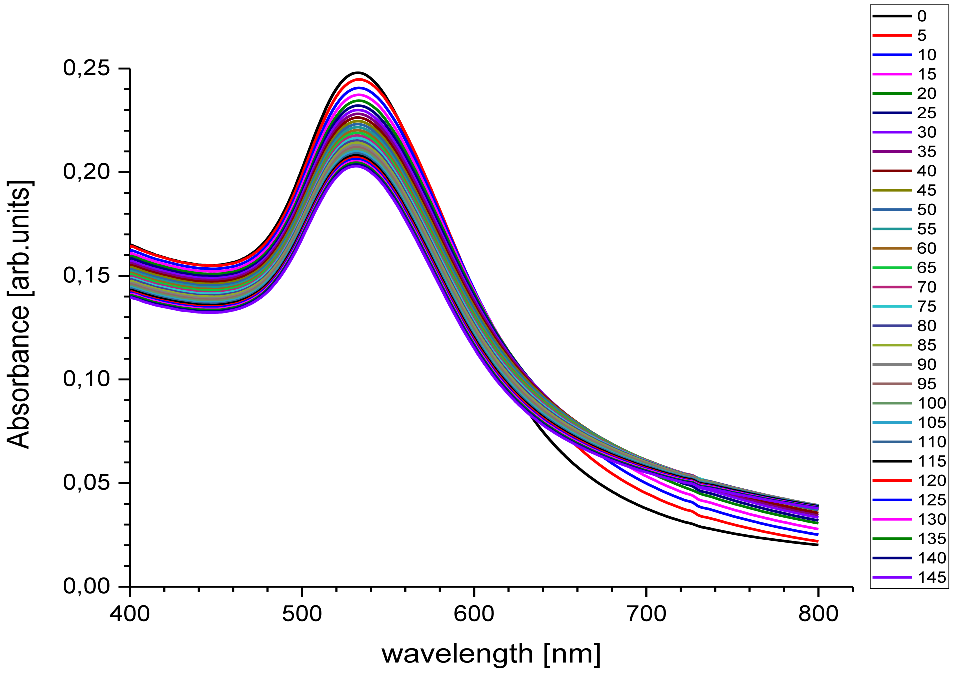

Figure S38: Time dependent absorption spectra of Au-AOT in diluted HCl at pH 3 and 100 mMol NaCl

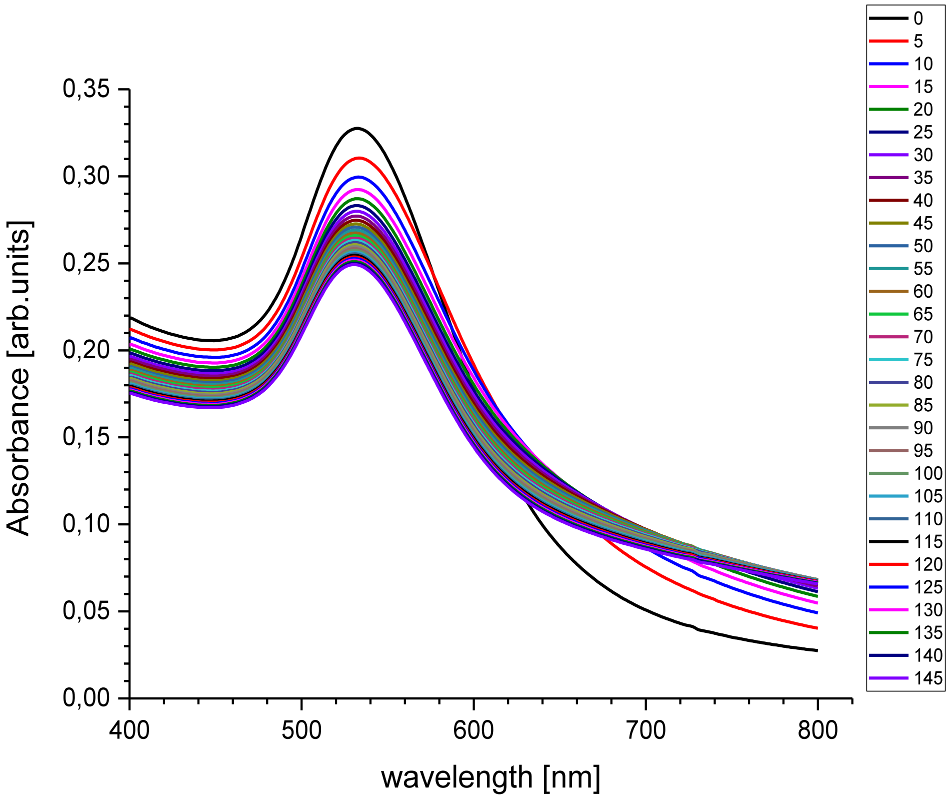

Figure S39: Time dependent absorption spectra of Au-AOT in diluted HCl at pH 3 and 125 mMol NaCl

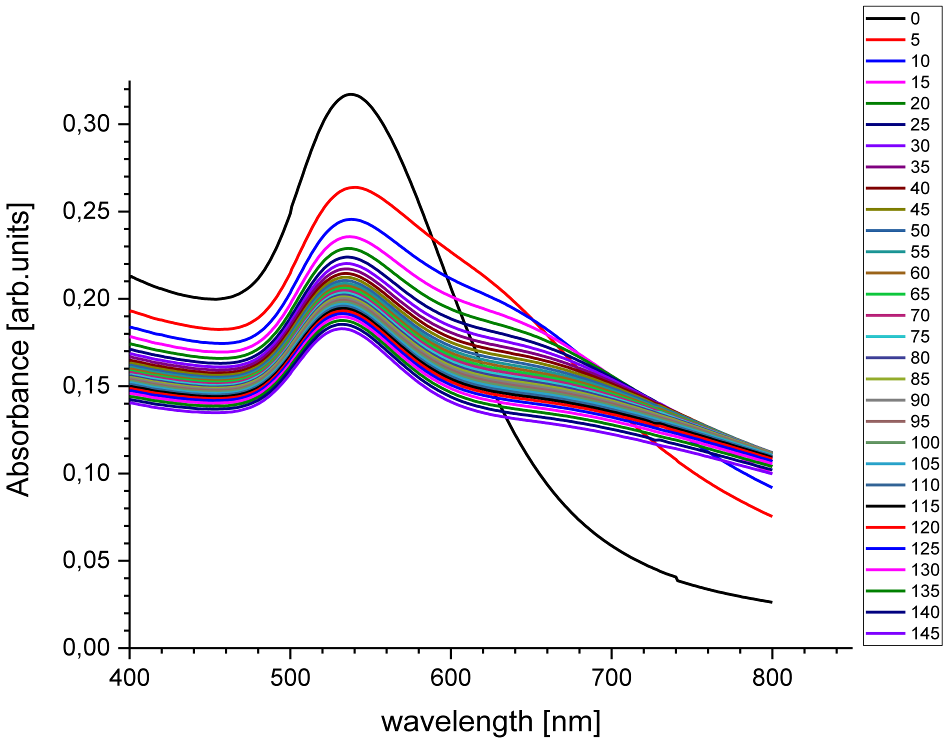

Figure S40: Time dependent absorption spectra of Au-AOT in diluted HCl at pH 3 and 150 mMol NaCl

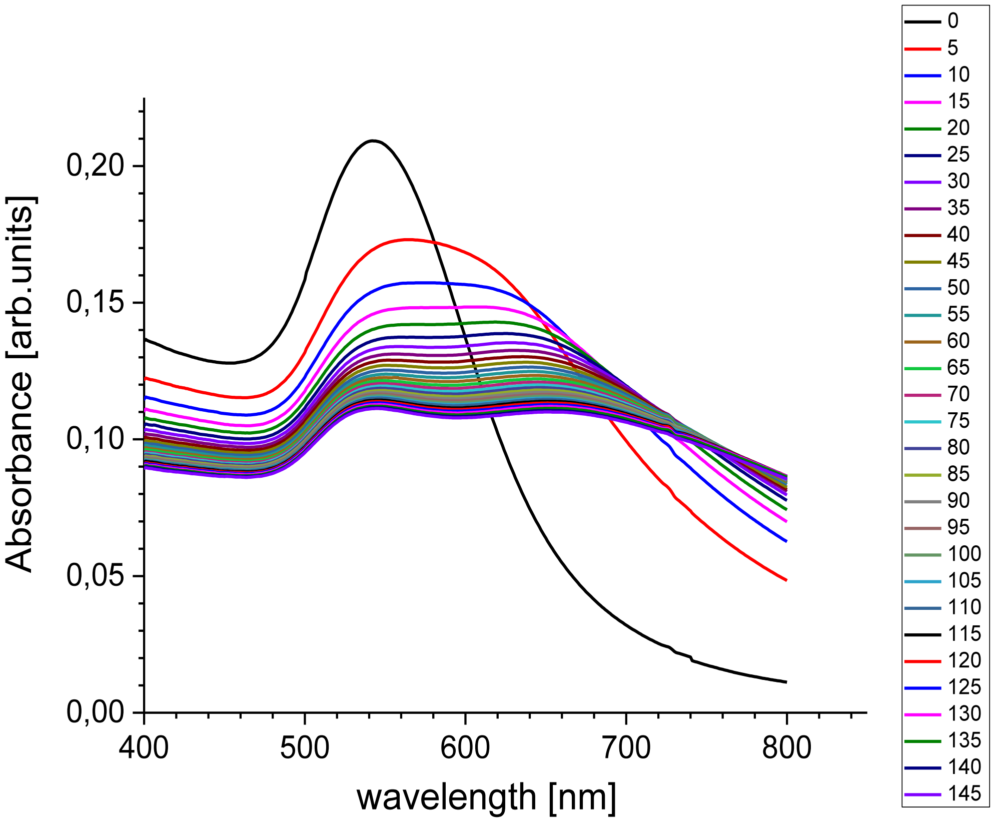

Figure S41: Time dependent absorption spectra of Au-AOT in diluted HCl at pH 3 and 175 mMol NaCl

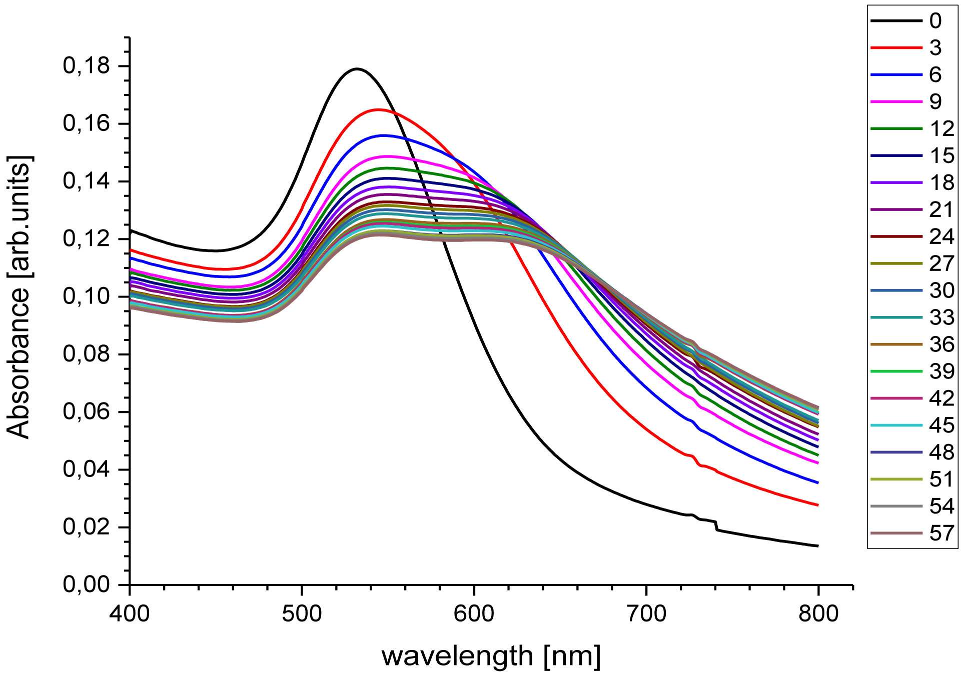

Figure S42: Time dependent absorption spectra of Au-AOT in diluted HCl at pH 3 and 200 mMol NaCl

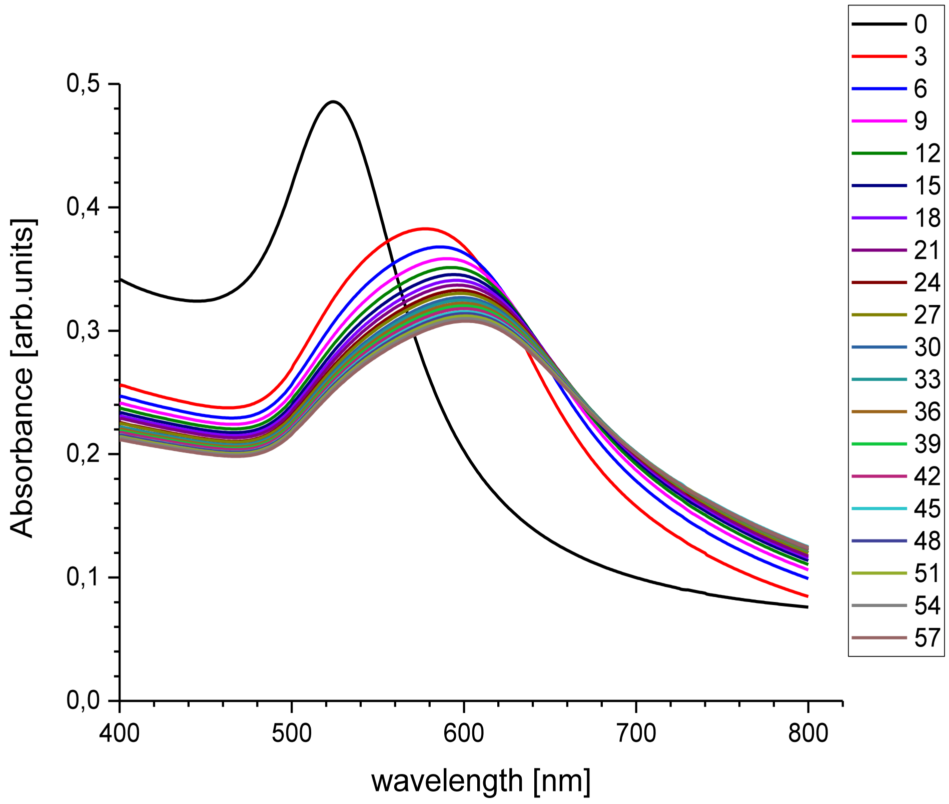

Figure S43: Time dependent absorption spectra of Au-AOT in diluted HCl at pH 3 and 225 mMol NaCl

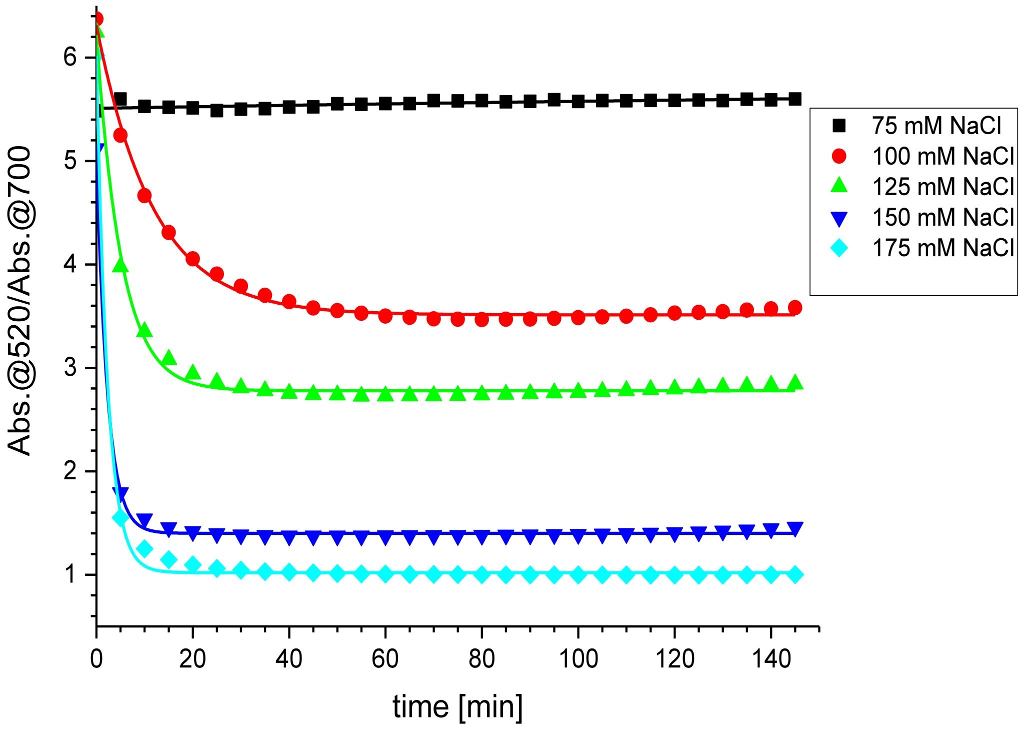

Figure S44: Progression of the ratio of absorbance *R* (symbols) for NaCl concentrations from 75 mM to 175 mM and the respective fit curves (lines) fitted by a first order exponential decay function.

12. Salt Induced Aggregation of Au-AOT with KCl

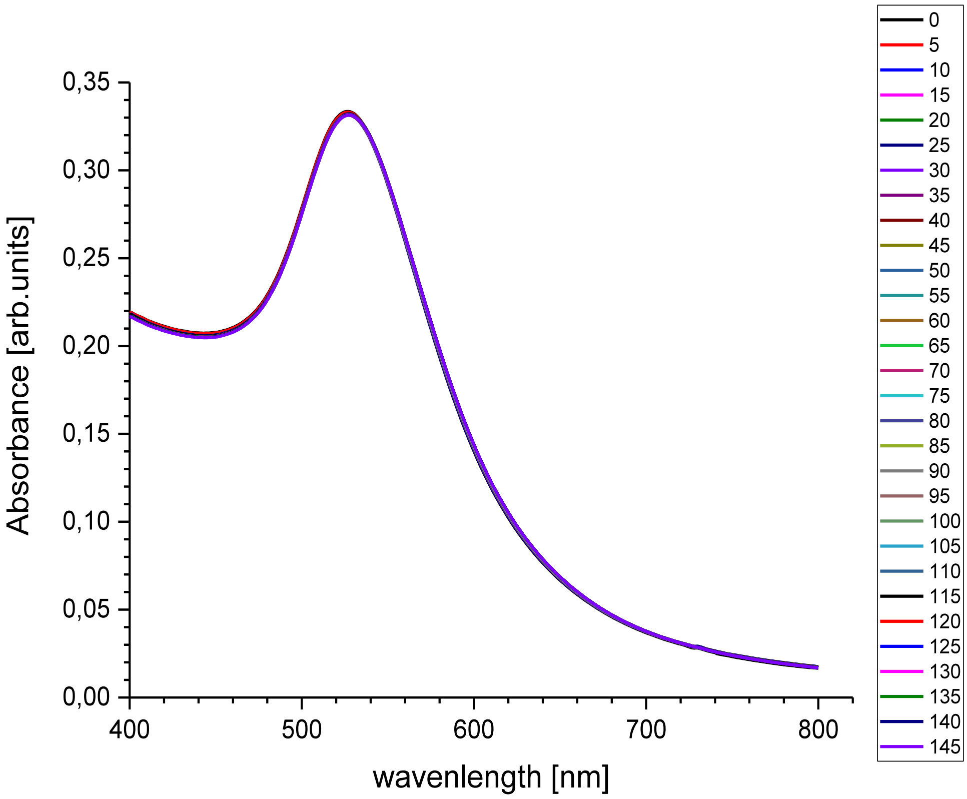

Figure S45: Time dependent absorption spectra of Au-AOT in diluted HCl at pH 3 and 100 mMol KCl

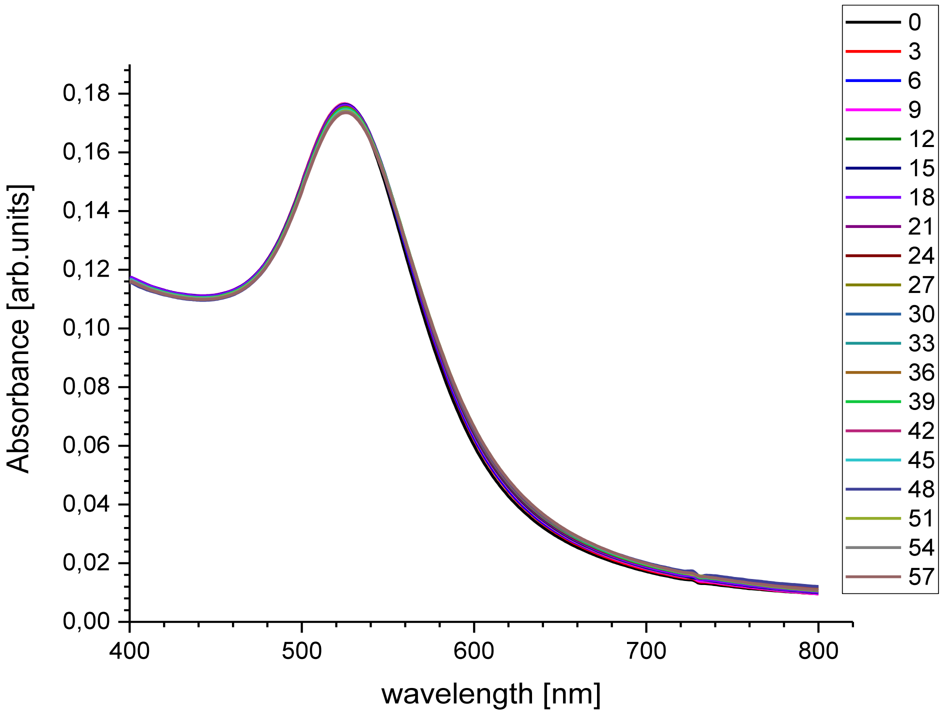

Figure S46: Time dependent absorption spectra of Au-AOT in diluted HCl at pH 3 and 125 mMol KCl

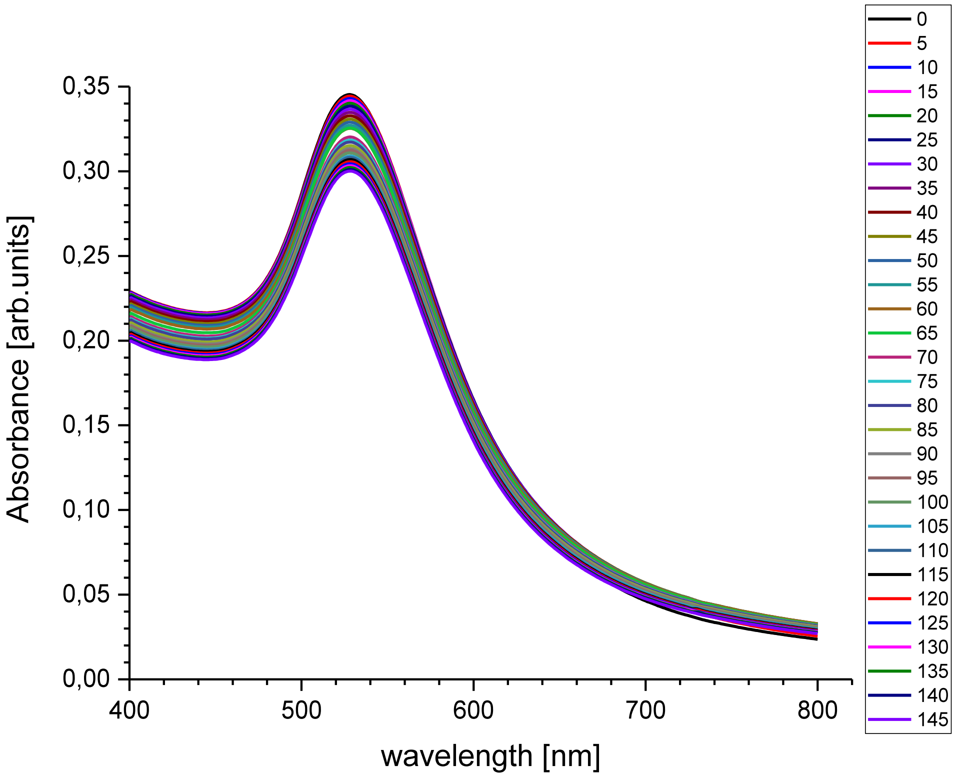

Figure S47: Time dependent absorption spectra of Au-AOT in diluted HCl at pH 3 and 150 mMol KCl

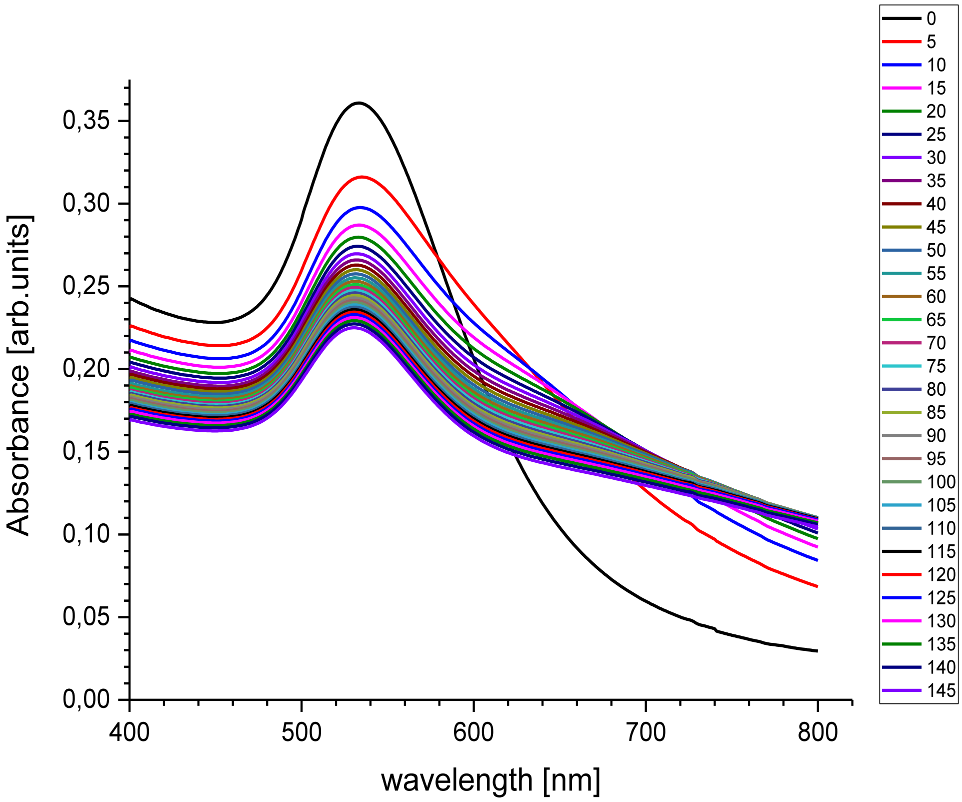

Figure S48: Time dependent absorption spectra of Au-AOT in diluted HCl at pH 3 and 175 mMol KCl

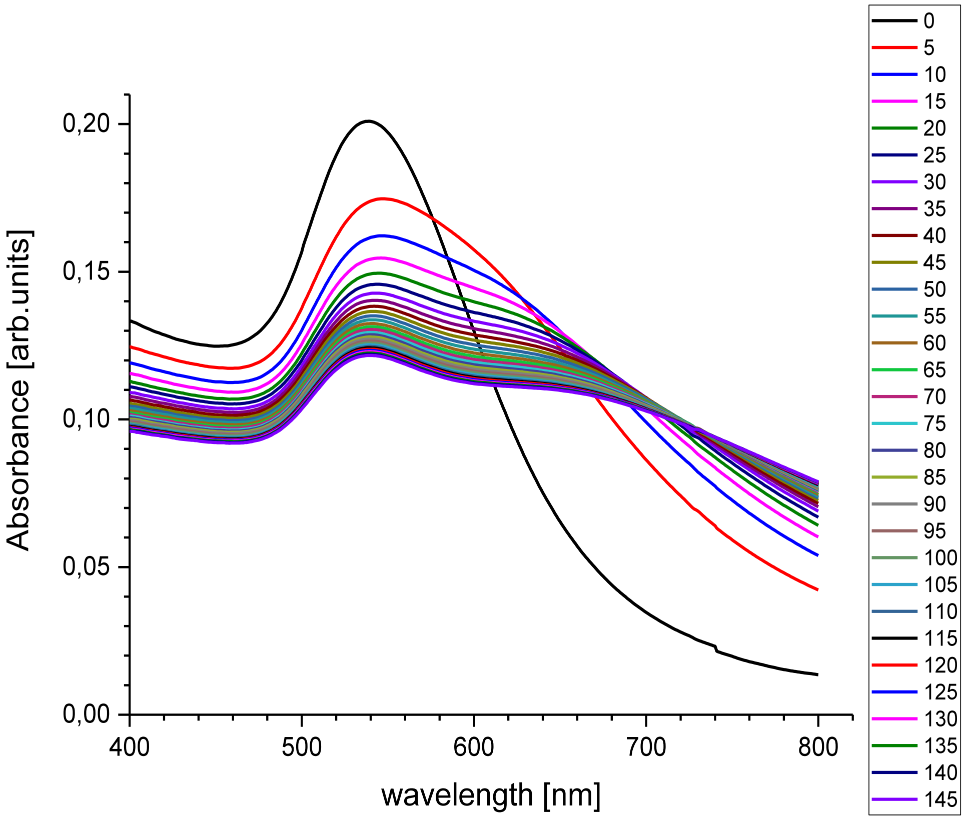

Figure S49: Time dependent absorption spectra of Au-AOT in diluted HCl at pH 3 and 200 mMol KCl

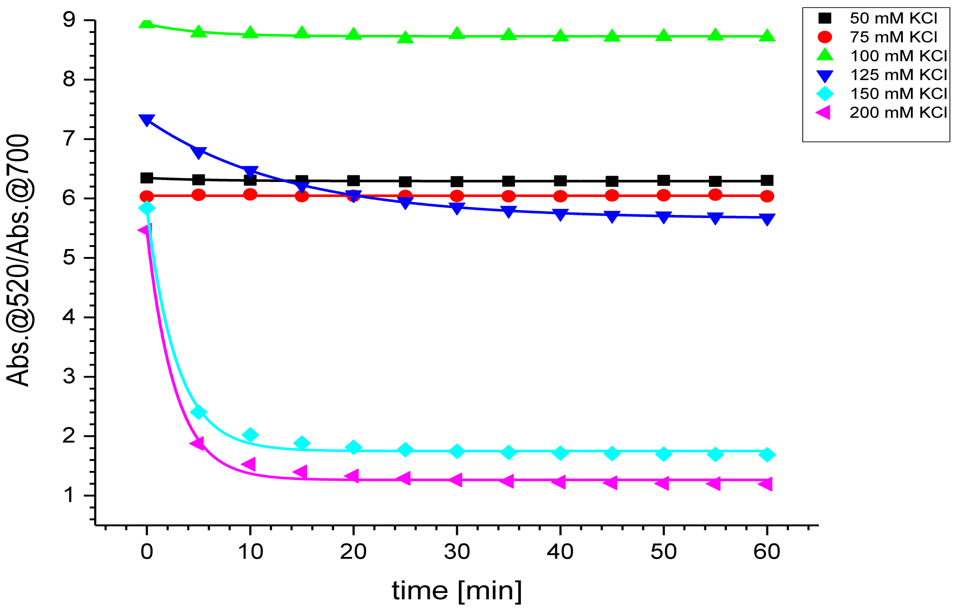

Figure S50: Progression of the ratio of absorbance *R* (symbols) for KCl concentrations from 50 mM to 200 mM and the respective fit curves (lines) fitted by a first order exponential decay function.

13. Salt Induced Aggregation of Au-AOT with CsCl

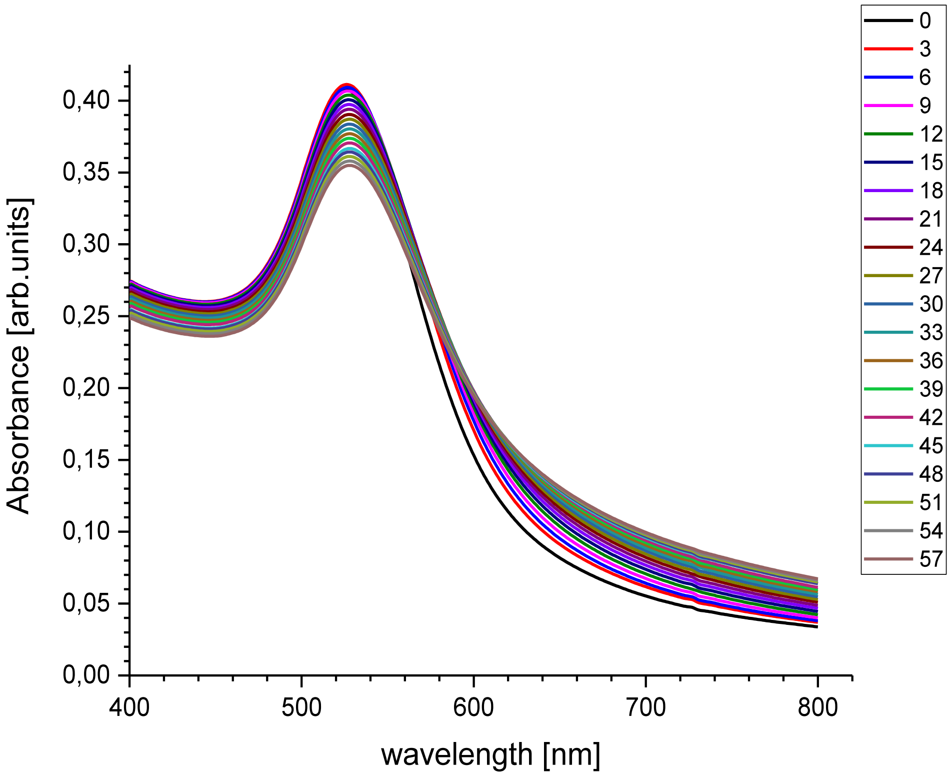

Figure S51: Time dependent absorption spectra of Au-AOT in diluted HCl at pH 3 and 200 mMol CsCl

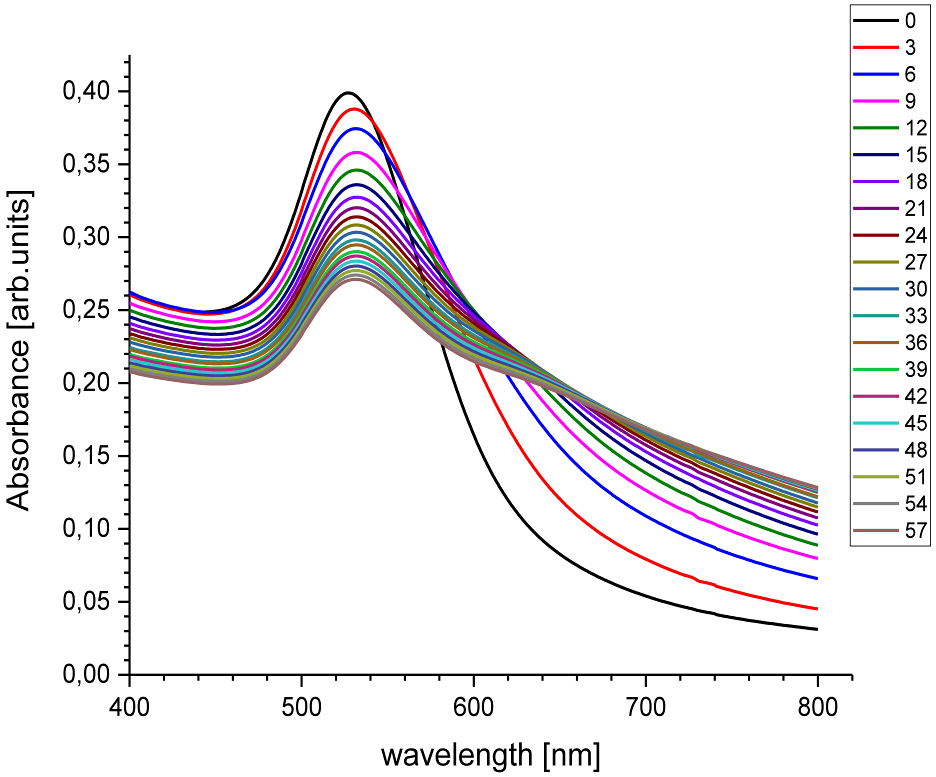

Figure S52: Time dependent absorption spectra of Au-AOT in diluted HCl at pH 3 and 225 mMol CsCl

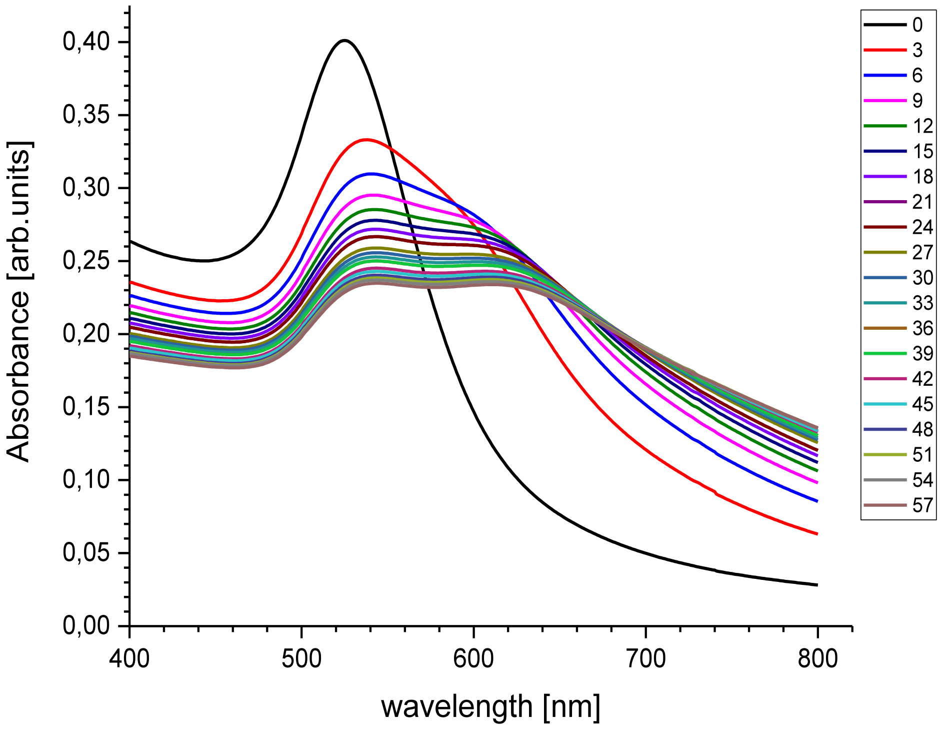

Figure S53: Time dependent absorption spectra of Au-AOT in diluted HCl at pH 3 and 250 mMol CsCl

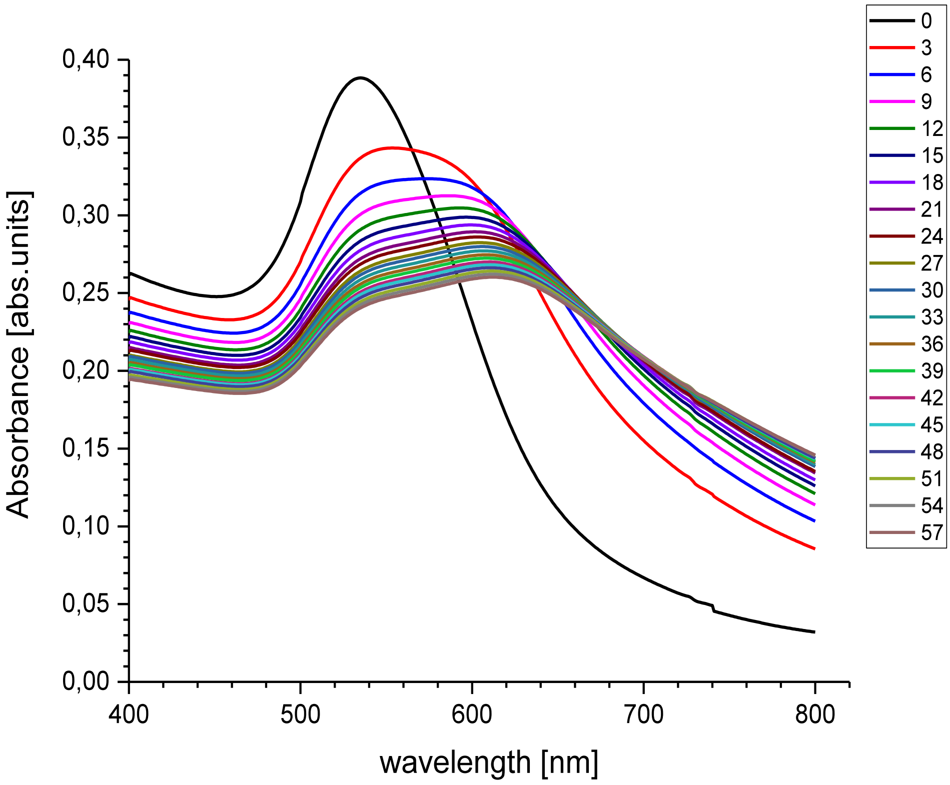

Figure S54: Time dependent absorption spectra of Au-AOT in diluted HCl at pH 3 and 275 mMol CsCl

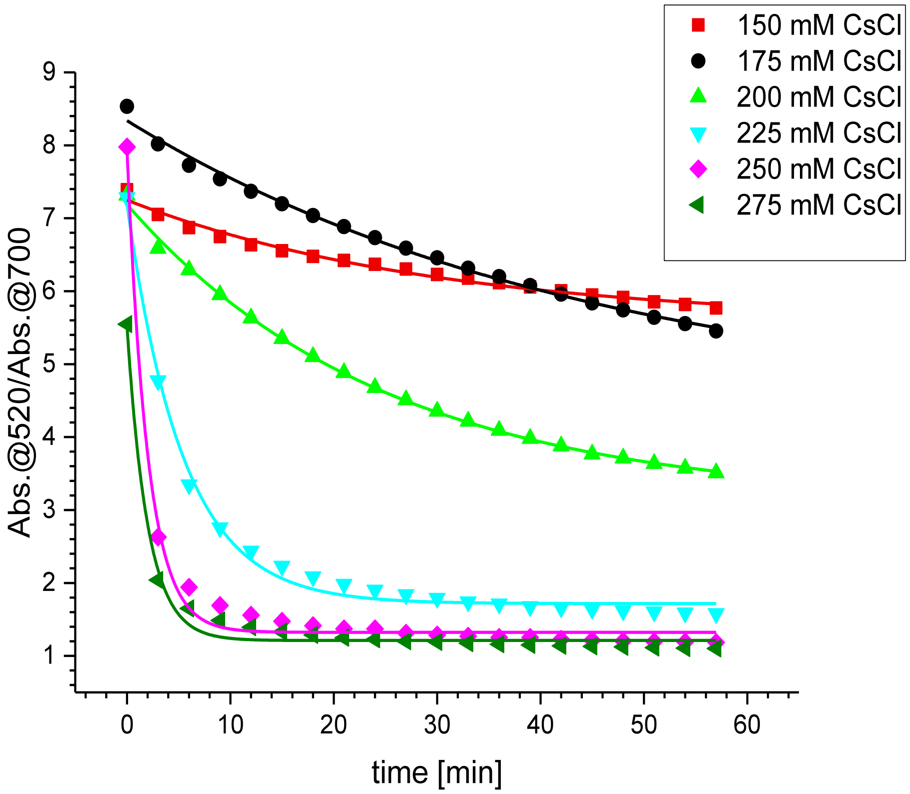

Figure S55: Progression of the ratio of absorbance *R* (symbols) for CsCl concentrations from 150 mM to 275 mM and the respective fit curves (lines) fitted by a first order exponential decay function.
